# Up to 16% Optical Carbon‐13 Hyperpolarization of Donor–Acceptor Molecules at 14.1 T

**DOI:** 10.1002/anie.7711870

**Published:** 2026-09-07

**Authors:** Marcel Levien, Máté Visegrádi, Ganesan Karthikeyan, Gilles Casano, Christian Reiter, Sebastian Wegner, Armin Purea, Michael R. Wasielewski, Federico De Biasi, Olivier Ouari, Lyndon Emsley

**Affiliations:** ^1^ Institut DES Sciences et Ingenierie Chimiques Ècole Polytechnique Fedérale DE Lausanne (EPFL) Lausanne Switzerland; ^2^ CNRS Institut DE Chimie Radicalaire Aix‐Marseille University Marseille France; ^3^ Bruker BioSpin GmbH & Co KG Ettlingen Germany; ^4^ Department of Chemistry Center for Molecular Quantum Transduction Institute for Quantum Information Research and Engineering Northwestern University Evanston Illinois USA; ^5^ Department of Chemical Sciences University of Padove Padova Italy

**Keywords:** donor–acceptor, NMR spectroscopy, optical hyperpolarization, photochemically induced dynamic nuclear polarization

## Abstract

Solid‐state nuclear magnetic resonance (NMR) is a powerful technique for structure determination with applications ranging from biology to material sciences. However, its inherent low sensitivity prevents many applications. In solids, optical hyperpolarization techniques such as solid‐state photochemically induced dynamic nuclear polarization (photo‐CIDNP) could alleviate this limitation, by generating nuclear spin hyperpolarization. We recently showed that ^1^H and ^13^C photo‐CIDNP can be observed for synthetic donor–acceptor (D–A) molecules. However, polarization levels in these systems are so far relatively low (<6%). In this study, we introduce a new D–A molecule for ^13^C solid‐state photo‐CIDNP, AnthraPol, which yields steady‐state signal enhancements of up to a factor −4500 at 14.1 T, corresponding to a nuclear polarization of ∼16%. We combine magnetic field dependent experiments and numerical simulations to demonstrate possible new rational design principles for solid‐state photo‐PAs, which guided the identification of AnthraPol as a suitable D–A system, and which led here to threefold improved signal enhancement over the previously reported CarboPol. By comparing bulk ^13^C enhancements, we also show that these local differences in polarization are directly reflected in the bulk signal enhancement.

## Graphical abstract

Up to 16% ^13^C nuclear polarization is achieved using a rationally designed photo‐CIDNP polarizing agent (PA) in magic‐angle‐spinning NMR experiments at 14.1 T. The PA was designed using a combination of theoretical photocycle calculations and magnetic field dependent experiments to identify important new design principles.

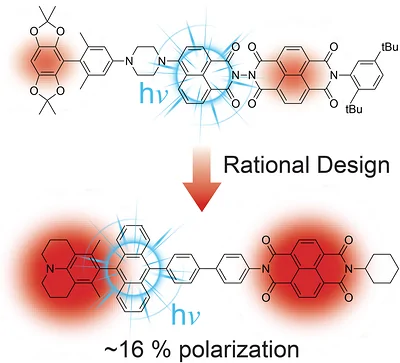

## 1 Introduction

Solid‐state nuclear magnetic resonance (NMR) spectroscopy is an important method for atomic‐level structure determination with applications to complex materials in areas ranging from biology to material science [1]. On the other hand, broader application of NMR is still hampered by its inherent low sensitivity which stems from the small energy difference between nuclear spin states.

To overcome this limitation, hyperpolarization methods have been investigated extensively [2, 3]. These techniques increase nuclear spin polarization by transfer of polarization from a more strongly spin polarized source to the nuclear spins of interest. In this context, optically induced hyperpolarization techniques are particularly promising, since they can in principle yield very high nuclear polarization levels [4, 5, 6, 7, 8, 9, 10, 11, 12], which would translate to a maximum ^1^H NMR signal enhancement of a factor *ε* ∼10400 at 9.4 T and 100 K. Among recently proposed avenues for optically induced nuclear spin hyperpolarization in solids [13, 14, 15, 16], photochemically induced dynamic nuclear polarization (photo‐CIDNP) is attractive as it can in theory generate polarization levels up to 100%, with experimentally observed enhancements reaching 20.000–80.000 at 1.4–2.4 T in biological systems [17]. It only requires irradiation with light, and can be active at various magnetic field strengths and at room temperature [18, 19, 20, 21]. Photo‐CIDNP can be observed in donor–acceptor (D–A) systems under light illumination when photo‐excitation is followed by charge separation to generate a transient spin‐correlated radical pair (SCRP, Figure 1) [21, 22, 23, 24]. If specific matching conditions are met [25, 26, 27, 28], then evolution and recombination of the SCRP can lead to the accumulation of hyperpolarization for hyperfine coupled nuclear spins [28, 29, 30].

**FIGURE 1:**
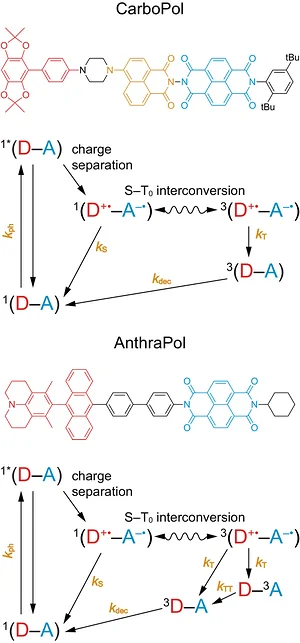
The chemical structures and photocycles of CarboPol (upper) and AnthraPol (lower). The benzobisdioxole aniline (BDX) donor, 4‐aminonaphthalene‐1,8‐dicarboximide (ANI) chromophore, and naphthalene‐1,8:4,5‐bis(dicarboximide) (NDI) acceptor in CarboPol are highlighted in red, yellow and blue, respectively. Bridge and end groups are displayed in black. *k*_ph_, *k*_S_, *k*_T_, and *k*_dec_ are the rates of photoexcitation, singlet decay, triplet decay and decay of the neutral triplet state, respectively. The combined donor and chromophore moiety 3,5‐dimethyl‐4‐(9‐anthracenyl)julolidine (DMJ‐An) and the acceptor NDI are highlighted in red and blue, respectively in AnthraPol. The photocycle of Anthrapol is similar to CarboPol with the exception that after the neutral triplet initially forms on the NDI moiety triplet‐triplet energy transfer occurs to the anthracenyl part of the donor with the rate *k*_TT_.

While solid‐state photo‐CIDNP was initially observed on ^13^C and ^15^N nuclei in biological systems [18, 21, 26, 31, 32, 33, 34, 35, 36, 37, 38, 39], we recently demonstrated that ^1^H and ^13^C spins can be polarized in synthetic D–A systems [40, 41, 42, 43]. Such systems are particularly promising as they could in principle be compatible with similar experimental workflows nowadays used in magic angle spinning dynamic nuclear polarization (MAS DNP). There, nuclear spin polarization is generated locally by polarization transfer from thermal electron spins [44, 45, 46, 47] that are added to the sample in the form of an exogeneous polarizing agent (PA), and where hyperpolarization is then spontaneously transferred from nuclei close to the PA to the bulk of the sample via spin diffusion [48, 49, 50, 51]. This approach makes MAS DNP a versatile technique with applications in many different fields.

We initially observed a solid‐state bulk ^1^H signal enhancement of *ε* = –16 via photo‐CIDNP at 0.3 T and 85 K [40], and subsequently identified differential relaxation (DR) as the dominant photo‐CIDNP mechanism [43]. To adapt the effect to much higher magnetic fields, we then targeted the three‐spin mixing (TSM) resonance condition [41]. Indeed, TSM is most efficient when *d* ≈ *ω_n_*, where *d* is the electron‐electron coupling in the SCRP and ω_n_ is the nuclear Larmor frequency [29, 30]. As *d* can easily be on the order of hundreds to thousands of MHz in D–A molecules [52], TSM appears well suited to generate large nuclear spin polarizations at high magnetic fields. For example, by reducing the D–A distance, the strength of *d* increases from –5.5 MHz in PhotoPol to around –560 MHz in PhotoPol‐S [7, 41, 53]. Using this latter D–A system we showed that the bulk ^1^H signal of *ortho*‐terphenyl (OTP)‐d_14_ (98.5% deuteration) can be enhanced by a factor of *ε* ≈ –100 at 9.4 T and 21.1 T [41]. This clearly demonstrated that targeting TSM is an attractive pathway to use photo‐CIDNP‐based PAs to hyperpolarize bulk solids at high magnetic fields. Intriguingly, the enhancements appeared to only have a weak magnetic field dependence using PhotoPol‐S.

The primary target nucleus for hyperpolarization at high magnetic field is ^1^H due to its high sensitivity, natural abundance and efficient spin diffusion. However, ^13^C photo‐CIDNP is also of high interest in this area since it can provide a more detailed probe of the photocycle kinetics and spin dynamics of the SCRP by directly observing the enhanced ^13^C signals of the D–A system [31, 32, 33, 39]. Doing this based on ^1^H effects can be quite challenging, because of spontaneous ^1^H‐^1^H spin diffusion that masks the ^1^H signal of the D–A molecule in favor of the creation of bulk hyperpolarization. As such, ^13^C photo‐CIDNP can be used as a powerful platform for testing rational design principles in these optical PAs.

In the context of small molecule PAs, we previously identified CarboPol (Figure 1), which has *d* ∼ –60 MHz, as a system suitable for ^13^C photo‐CIDNP at 9.4 T (^13^C Larmor frequency of *ω_n_* 100 MHz) via the TSM mechanism [42, 53], and found that it indeed yielded signal enhancements of |*ε*| > 2300 on some of the ^13^C sites of the D–A molecule itself (for a recycle delay of 1.8 s) [42]. While these results are very encouraging, the polarization levels are still relatively modest, and the photo‐CIDNP polarization build‐up times (*T*_b_) are slow.

Here, by using numerical simulations to predict which factors are more likely to affect the overall ^13^C photo‐CIDNP performance in CarboPol [54], we introduce another photo‐PA, dubbed AnthraPol, (Figure 1) which has a factor of two longer SCRP lifetime in liquids at room temperature [53, 55]. Experimentally, we find that the overall ^13^C polarization is approximatively a factor of three larger for AnthraPol than for CarboPol at 9.4 T. Using the thermalized ^13^C signal from bulk OTP as an internal reference, we estimate the steady‐state signal enhancement of the different ^13^C sites on the photo‐PAs. We report enhancements reaching *ε* = –4470 ± 895 for AnthraPol at 9.4 and 14.1 T, corresponding to a ^13^C nuclear spin polarization of ∼10% and ∼16%, respectively. We also show that such improvements of local polarization can be translated to larger bulk ^13^C signal enhancements obtained via ^13^C‐^13^C spin diffusion, even though the process is strongly hampered under MAS conditions. By analyzing the magnetic field dependence of the two photo‐PAs, we experimentally demonstrate that the magnetic field dependence for TSM‐driven photo‐CIDNP is not necessarily dictated solely by the matching condition but might also depend on parameters such as Δ*ω*_e_ [54]. Indeed, comparing enhancements to experimental *d* and *g*‐values, as well as the kinetic rates of the photocycle, we experimentally confirm design principles for efficient donor–acceptor systems recently proposed from numerical predictions [54].

## 2 Results and Discussion

CarboPol (Figure 1) is composed of a benzobisdioxole aniline (BDX) donor, 4‐aminonaphthalene‐1,8‐dicarboximide (ANI) chromophore and a naphthalene‐1,8:4,5‐bis(dicarboximide) (NDI) acceptor. For clarity, the donor, chromophore and acceptor (D‐C‐A) of CarboPol will be denoted as BDX‐ANI‐NDI throughout. After absorption of light, the excited state undergoes rapid intramolecular electron transfer to yield the singlet‐born SCRP (see Figure 1) [4]. Because the singlet state is not an eigenstate of the high field Hamiltonian, the SCRP coherently evolves between the singlet state |S〉, denoted as [1] (BDX^+•^–ANI–NDI^–•^), and triplet state |T_0_〉, denoted as [3] (BDX^+•^–ANI–NDI^–•^), of the SCRP. Nuclear hyperpolarization can be generated during the evolution of the SCRP and/or after its recombination, if certain conditions are met. The smallest spin system for this process consists of two electron spins (*S* = 1/2) and one nuclear spin (*I* = 1/2), which couples to the electron spins via hyperfine coupling [29, 30]. Because in the D–A systems studied here the SCRP is initially generated in the singlet state and can evolve coherently only to |T_0_〉, we can neglect the |T_+_〉 and |T_−_〉 states and consequently simplify the analysis to a pseudo‐two spin system [28]. The two spin Hamiltonian that describes the photo‐CIDNP process in the high magnetic field limit is then given by [28]:

(1) $$\;\hat{H}=\Delta \omega_{e}\;{\hat{S}}_{z}+\omega_{n}{\hat{I}}_{z}+d{\hat{S}}_{x}+a{\hat{S}}_{z}{\hat{I}}_{z}+b{\hat{S}}_{z}{\hat{I}}_{x}$$

where Δ*ω*_e_ = (*ω*_1e_−*ω*_2e_) is the difference of the Larmor frequencies of the two electron spins, *ω*_n_ is the nuclear Larmor frequency and *d* the electron–electron (e–e) coupling, given by *d* = –2*J*_ex_−*D*. Here, *J*_ex_ and *D* are the exchange and the dipolar contributions, respectively, to the e‐e interaction. Finally, *a* and *b* are the secular and pseudo‐secular components of the hyperfine coupling.

For TSM, the most favorable photo‐CIDNP mechanism at high magnetic field, nuclear hyperpolarization is generated via coherent spin flips (e.g., |Sα_*n*_〉↔|T_0_β_*n*_〉) within the SCRP. TSM is maximized under the following matching condition [54]:

(2) $$\;\left|\omega_{n}\right|=\frac{1}{2}\;\sqrt{d^{2}+{\left(\Delta \omega_{e}+\frac{a}{2}\right)}^{2}}+\frac{1}{2}\sqrt{d^{2}+{\left(\Delta \omega_{e}-\frac{a}{2}\right)}^{2}}$$

which simplifies to |*ω*_n_| ≈ |*d*| for a *d* value much larger than Δ*ω*_e_ and *a*. Ultimately, as depicted in the top part of Figure 1, the SCRP decays via the singlet or triplet channel with *k*_S_ and *k*_T_, respectively. While the singlet state directly decays to the ground state, the triplet decays first to a neutral triplet state (BDX–ANI–^3^NDI), for CarboPol localized on the NDI [56], which subsequently decays to the ground state with *k*_dec_.

With the objective of obtaining higher polarization levels and faster build‐up times, we recently performed a numerical analysis of the spin dynamics occurring during the photocycle, with a focus on the TSM mechanism [54]. We did this by extending the established framework [28, 29, 30, 57, 58] to the full photocycle [54]. Notably, we found that satisfying the matching condition *d* ≈ *ω*_n_ not only boosts the maximum achievable polarization level, but also reduces the polarization build‐up time *T*_b_. Similarly, the pseudo‐secular hyperfine coupling *b* acts as a driving force for TSM, with larger *b* values favoring high polarization and short *T*_b_. This is in agreement with previous theoretical treatments of solid‐state photo‐CIDNP [27, 28]. Additionally, long SCRP lifetimes, small Δ*ω*_e_, as well as a faster charge recombination rate via the triplet channel than for the singlet channel (i.e., *k*_S_<*k*_T_) are predicted to favor efficient hyperpolarization for a singlet‐born SCRP [54].

To test these theoretical predictions, we identified the structure of AnthraPol from a large family of previously reported D–A molecules [55, 59, 60, 61, 62, 63] (similarly to the pool that CarboPol was drawn from [4, 7, 10, 52, 53, 56, 64]). It was chosen because of its smaller Δ*ω*_e_, as well as more favorable kinetic parameters (*k*_S_<*k*_T_) and a factor of two longer SCRP lifetimes as compared to CarboPol at room temperature [55]. Even though *d* is known for AnthraPol only in solution at room temperature (*d* ≈ 840 MHz),[55] *d* ≈ 130 MHz at 85 K in toluene was measured for a similar D–A molecule with the same donor and acceptor but with a ∼2.8 Å bridge between the two moieties [59].

The photocycle of AnthraPol, which consists of a 3,5‐dimethyl‐4‐(9‐anthracenyl)julolidine (DMJ‐An) donor and naphthalene‐1,8:4,5‐bis(dicarboximide) (NDI) acceptor, is slightly different. We denote the donor and acceptor of AnthraPol as (DMJ‐An–NDI). In analogy with CarboPol, after triplet decay, the neutral triplet is predominantly formed on the NDI moiety (DMJ‐An–^3^NDI). However, this neutral triplet is then converted with the rate *k*_TT_ to a second neutral triplet state localized on the anthracenyl part of the donor (DMJ‐^3^An‐NDI), which then decays to the ground state [63]. As we will discuss below, these differences in the photocycle also influence significantly the observed signal enhancements.

All experiments were performed with sample temperatures of ~100 K in 3.2 mm sapphire rotors at 8 kHz MAS. Light irradiation at 9.4 T was achieved with a low‐temperature MAS DNP probe, which was modified to accommodate a permanently installed optical fiber (400 µm diameter). The end of the optical fiber is inserted from the bottom plate into the gas channel that controls the sample temperature. A mirror then directs the light beam onto the sample (see Figure S2). The distance between the end of the fiber and the sample is ∼16.5 mm. This approach enables straightforward irradiation of the sample without compromising any VT, radiofrequency or microwave capabilities in the probe. More details on the probe assembly and the performance are given in the Section S2. Experiments at 11.7 T, 14.1 T and 21.15 T were performed using the previously established approach of inserting an optical fiber (400 µm diameter) through the microwave waveguide of LTMAS DNP probes (14.1 T and 21.15 T) [41] or by using an additional opening at the probe base (11.7 T).

1.5 mM and 2.25 mM solutions of CarboPol and AnthraPol, respectively, in OTP were degassed by freeze‐pump‐thaw cycles inside the 3.2 mm rotor with a vacuum of ∼10^−2^ mbar. As OTP is a solid at room temperature, the sample was heated above 65°C during each cycle. In between cycles, OTP was crystallized in the rotor, which improved O_2_ removal efficiency and led to increased *T*_1n_ values. At least three cycles were performed for each sample or until no air bubbles were observed during the melting of the sample. Figure 2a,b shows the laser‐on (∼1.2 W output power, at a wavelength of 450 nm) spectra of CarboPol and AnthraPol, respectively, obtained with a polarization delay of 20 s. Note that signal enhancements of the ^13^C sites of the photo‐PAs cannot be measured directly, because they are below the limit of detection in the laser‐off experiments. Hence, they were evaluated by using the ^13^C resonance of OTP at long recycle times (12500 s) without light irradiation as an internal reference. Since *T*_b_ of the ^13^C nuclei on the photo‐PAs is ≲ 25 s and *T*_1n_ of ^13^C of OTP is ≥ 1000 s (see Figures S4 and S5), we can assume that any signal observed using 20 s recycle times under continuous laser irradiation mainly stems from the photo‐PA, while at 12500 s and without laser irradiation, we exclusively observe the thermalized OTP signal. Details on the enhancement calculation are given in the Section S3. Nevertheless, as we observe a minor contribution of OTP to the ^13^C spectrum of CarboPol at 20 s (see Figure 2a), the enhancements calculated using this approach may be subject to a larger uncertainty than direct comparison of laser‐on and off experiments. We therefore assign an uncertainty of 20% to the signal enhancements obtained from internal referencing with OTP.

**FIGURE 2:**
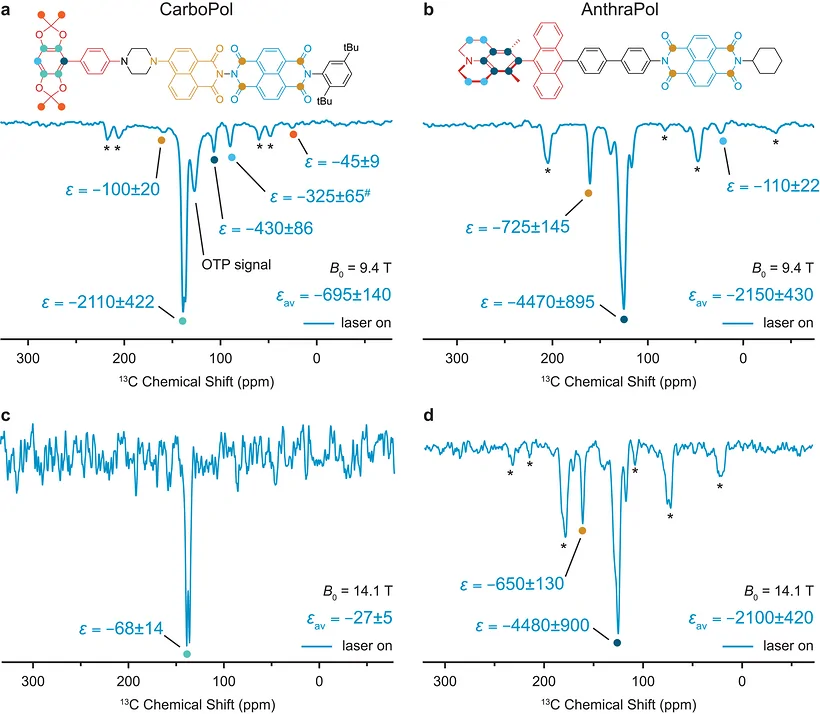
(a) ^13^C NMR spectrum of a 1.5 mM frozen solution of CarboPol in OTP under continuous light irradiation (450 nm, ∼1.2 W output laser power), at 9.4 T, with a sample temperature of 100 K and with 8 kHz MAS. The polarization/recycle delay between scans was set to 20 s to ensure that almost no OTP signal was observed. The colored dots on the resonances indicate the assignment to the different ^13^C nuclei indicated on the chemical structure of CarboPol shown above the spectrum. (b) ^13^C NMR spectrum of a 2.25 mM frozen solution of AnthraPol in OTP under identical experimental conditions as in (a). (c) ^13^C NMR spectrum of a 1.5 mM frozen solution of CarboPol in OTP at 14.1 T. All other parameters were identical to (a). (d) ^13^C NMR spectrum of a 2.25 mM frozen solution of AnthraPol in OTP at 14.1 T. All other parameters were identical to (b). The assignments are explained in the text. More experimental details are given in the Supporting Information. (^#^) We note that the enhancement of this site in (a) might be slightly overestimated due to a longer *T*_B_ ≈ 22 s, however longer recycle delays would reduce the suppression of the OTP signal and therefore induce larger errors on the overall enhancement estimation. The asterisks denote spinning sidebands.

Figure 2a shows the laser‐on spectrum with a polarization delay of 20 s for a frozen solution of 1.5 mM CarboPol in OTP at 9.4 T. Using the previously established assignment of the resonances [42], we were able to determine the steady‐state enhancement of all hyperpolarized ^13^C resonances reaching up to a factor of *ε* = –2110 ± 422 for the ^13^C nuclei in the aromatic system of the BDX moiety. This corresponds to a polarization level of 5%. We were also able to determine an average enhancement of all hyperpolarized carbons (the 14 carbons highlighted in Figure 2a) of *ε* = –695 ± 140. In contrast, Figure 2b shows the laser‐on experiment for AnthraPol with a polarization delay of 20 s. On average we obtain an enhancement of *ε* = –2150 ± 430 for the hyperpolarized carbons of AnthraPol.

More specifically, we determine the enhancement factor of the main peak in the AnthraPol spectrum shown in Figure 2b to be *ε* = −4470 ± 895 corresponding to a polarization of ∼10% and easily exceeding the theoretical limit for microwave induced DNP at this magnetic field and 100 K. For this, we first note that no spin density is localized on the anthracene moiety of the donor [55], which allows to exclude those carbons from our analysis. Using the same argument, we also neglect the two phenyl bridges. Even though it is unlikely, it is also possible that the aromatic carbons of the NDI contribute to the main peak. Indeed, in AnthraPol the neutral triplet state is localized on the An moiety, hence the NDI signals could be less quenched by paramagnetic relaxation enhancement (PRE) effects, as opposed to CarboPol. An estimation of the enhancement of these ten carbons is given in Supplementary Note S1 and could reduce the enhancement of the main peak by up to a factor of 800, which is still within the estimated uncertainty of the enhancement.

Then, we can assign the peaks at ∼21 ppm to the four methylene groups of the DMJ donor, which are enhanced by a factor of *ε* = −110 ± 22. Furthermore we see that the methyl groups as well as the methylene groups neighboring the nitrogen atom on the DMJ donor (∼50 ppm) are not enhanced by photo‐CIDNP (corroborated by a laser on spinning sideband suppression (TOSS) experiment [65, 66], Figure S6).

We can furthermore assign the resonance at ∼160 ppm to the four carbonyl carbons of the NDI. Interestingly, comparing the enhancement of the same moiety between CarboPol and AnthraPol reveals that the enhancement on the NDI carbonyls is increased by a factor of seven for AnthraPol, that is, from *ε* = −100 ± 20 to *ε* = −725 ± 145. This stark difference can be attributed to the fact that the neutral triplet state is localized on the NDI for CarboPol, while it is converted to a neutral triplet state localized on the An moiety for AnthraPol [63]. As a consequence, PRE induced by the triplet state severely reduces the polarization accumulated via TSM on the NDI for CarboPol. This effect is reduced for AnthraPol resulting in a larger signal enhancement of the NDI carbonyl carbons. Considering that the lifetime of BDX–ANI–^3^NDI is only ∼60 µs at 85 K in butyronitrile (PrCN) [53], while the neutral triplet is localized for ∼10 µs on the NDI in AnthraPol (at 85 K in toluene) [63], such a detrimental effect of the PRE on the hyperpolarization is somewhat surprising and should be considered in the design of new photo‐PAs with increased efficiency.

To investigate the magnetic field dependence of CarboPol and AnthraPol, in addition to the 9.4 T measurements, we performed the same set of experiments at 11.7, 14.1 and 21.15 T. Figure 2c,d shows that while increasing the magnetic field to 14.1 T reduces the maximum enhancement for CarboPol to *ε* = –68 ± 14, the enhancement for AnthraPol is the almost identical to that at 9.4 T, reaching *ε* = –4480 ± 900 for the aromatic carbons of the donor. At 14.1 T this corresponds to a nuclear polarization of ∼16%.

To further analyze the magnetic field dependence, we show the average nuclear polarization of CarboPol and AnthraPol as a function of the magnetic field in Figure 3a. The field dependence is distinct for the two photo‐PAs. While CarboPol shows a steady decline toward high magnetic field, AnthraPol increases up to 14.1 T, reaching an average of ∼8%, before decreasing to ∼3% at 21.15 T. To explain these observations, we calculated the full matching condition (eq.2) for CarboPol and AnthraPol using the parameters given in Table 1. Figure 3b shows the matching condition as a function of the magnetic field for CarboPol and AnthraPol as well as the ^13^C nuclear Larmor frequency. The intersections between CarboPol and AnthraPol with *ω*_n_ indicate the optimum magnetic field. Strikingly, for CarboPol this is around 14 T, a magnetic field, where we experimentally determined almost no ^13^C photo‐CIDNP effect.

**FIGURE 3:**
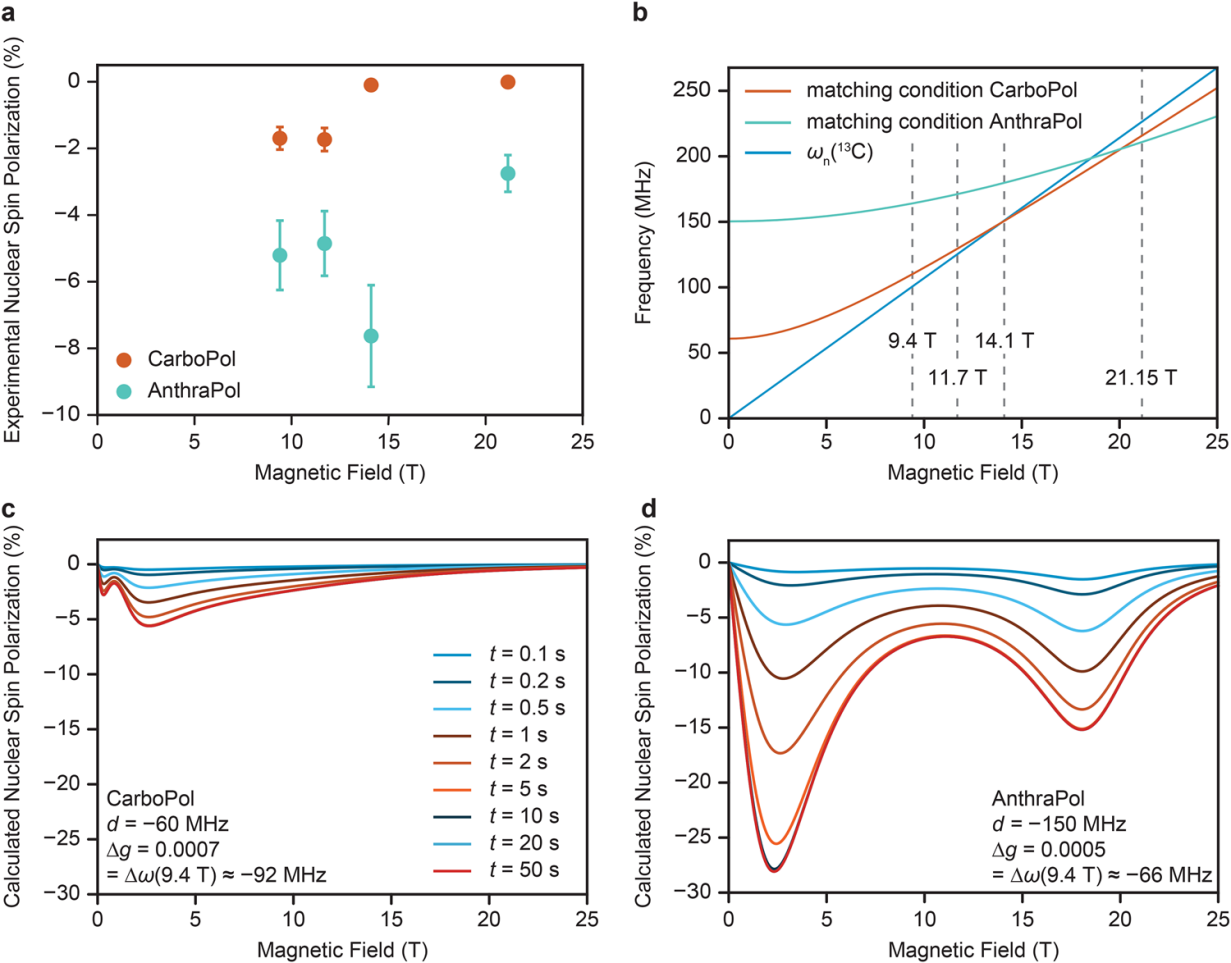
(a) Experimental average nuclear spin polarization of all hyperpolarized carbon‐13 as a function of the magnetic field. CarboPol and AnthraPol are displayed in orange and turquoise, respectively. (b) Predicted matching condition for Carbopol and AnthraPol, respectively, and carbon‐13 Larmor frequency as a function of the magnetic field. Matching conditions were calculated using eq.2 and an estimated *a* = 20 MHz, while Δ*g* = 0.0007 and *d* = −60 MHz and Δ*g* = 0.0005 and *d* = −150 MHz were used for CarboPol and AnthraPol, respectively. (c, d) Predicted nuclear spin polarization as a function of the magnetic field using our recently introduced kinetic photocycle treatment based on the Liouville von Neumann equation $\frac{d}{dt}\;\tilde{\rho }=i\tilde{H}\tilde{\rho }-\tilde{R}\tilde{\rho }$ and $\tilde{\rho }\;\left(t\right)=\exp\left[\left(-i\tilde{H}+\tilde{R}\right){\tilde{\rho }}_{0}\right]$ [54]. We used the same parameters in (c, d) with the exception of *d* and Δ*g* (Δ*ω*_e_), which are given in the insets. Other parameters were chosen according to the example data set used in our recent study: *k*_dec_ = 10^1^ s^−1^, *k*_exc_ = 10^2^ s^−1^, *k*_s_ = 2.22 × 10^7^, *k*_T_ = 2.0 × 10^8^ s^−1^, *T*_1gs_ *=* 5 s, *T*_1nT_ *=* 1 s, *a* = 20 MHz and *b =* 5 MHz [54].

**TABLE 1:** Summary of parameters used in the calculation of matching conditions and full photocycle simulations.

Photo‐PA	Δ*g*	Δ*ω*_e_ at 9.4 T (MHz)	*d* (MHz)
CarboPol	0.0007 [7, 53, 67]	∼92	−60 [42]
AnthraPol	0.0005 [63, 67]	∼66	−150^a^

^a^ *d* is reported to be ∼130 MHz at low temperature only for a molecule with identical donor and acceptor but with a ∼2.8 Å bridge (additional alkyne group) [59]. We therefore assume a slightly larger *d* for AnthraPol.

One way to explain this quite intriguing observation could be to employ full photocycle calculations based on the Liouville master equation [54]. Figure 3c,d shows the calculated magnetic field dependence of the nuclear polarization using the Δ*g* and *d* specific to CarboPol and AnthraPol (Table 1), respectively. All other parameters involved in the calculation were the same and were taken from an example parameter set employed in our recent publication (see caption of figure 3) [54]. Without fitting any of the involved parameters, we find that the full photocycle calculations qualitatively reproduce the experimentally observed trends. Considering the uncertainty in many of the kinetic and spin chemistry parameters, this agreement is quite striking. Indeed, the model predicts a monotonic decay of polarization for CarboPol at high magnetic field, which can be explained by the detrimental effect of larger Δ*ω*_e_ at higher magnetic fields that strongly reduce the TSM efficiency (even if the matching condition in eq.2 is formally satisfied). In the case of AnthraPol, two maxima are observed. The peak at low field is likely caused by DR, while the high field peak is caused by TSM. All of these observations are in line with our experimental observations, although we note that all the kinetic rates as well as hyperfine couplings are unknown and therefore these calculations are likely to describe only rough trends. Nevertheless, additional calculations using Δ*g* = 0.0005 (experimental value for AnthraPol) and *d* = –60 MHz (experimental value for CarboPol, Figure S10) show that the TSM contribution is recovered at lower magnetic field. Vice versa, using Δ*g* = 0.0007 and *d* = –150 MHz, we find full suppression of the TSM contribution at high field (Figure S11). We thus see in the simulations that TSM is suppressed as soon as Δ*ω*_e_ becomes comparable in size to *d*. This is in line with earlier calculations that showed that large Δ*ω*_e_ prevent efficient mixing of states for TSM [54].

While these calculations suggest that Δ*ω*_e_ plays an important role in the different performance of CarboPol and AnthraPol, other parameters such as the hyperfine coupling and the kinetic rates might still also contribute and will be discussed here.

There is little experimental data for the pseudo‐secular hyperfine couplings (*b*) in these systems, though differences in *b* are expected to have a significant effect on the enhancements. Experimentally we do see measurable enhancements on the carbons that are predicted to have larger isotropic hyperfine couplings [7].

Additionally, we predict the kinetic parameters of the photocycle may play a crucial role in the performance of CarboPol and AnthraPol [54]. Indeed, the favorable ratio of *k*_S_ and *k*_T_ at room temperature ($\frac{k_{T}}{k_{S}}\approx 2.2$) [55] and the two‐fold longer SCRP lifetime at room temperature compared to CarboPol [53, 55], were among the main motivations to select AnthraPol as a possible photo‐PA [55]. If these trends prevail at low temperature, both factors will favorably affect the signal enhancement of AnthraPol. On the other hand, there is no data for *k*_S_ and *k*_T_ for CarboPol at high or low temperature.

Finally, the different overall efficiencies between CarboPol and AnthraPol can be propagated to the enhancements observed for the ^13^C signals of bulk OTP. As pointed out above, even though inefficient, spontaneous ^13^C–^13^C spin diffusion can still be observed in these samples due to the very long *T*_1n_ of the ^13^C nuclei of OTP. By comparison with literature data, and assuming a *T*_1_ of ∼1000 s for the ^13^C nuclei of OTP, we previously estimated a spin diffusion length of $\sqrt{DT_{1n}}=\lambda =100$ nm for a static sample [42]. Here, *D* is the spin diffusion coefficient and λ the spin diffusion length. This length is likely to be reduced under MAS, due to partial averaging of the dipolar interactions [68]. Figure 4 shows the laser on and off experiments with a recycle delay of 5000 s at 9.4 T and 14.1 T. We obtain bulk ^13^C signal enhancements of the OTP signals of *ε* = –11 ± 1 and *ε* = –33 ± 1 at 9.4 T using CarboPol and AnthraPol, respectively (the large difference in absolute value between local ^13^C enhancements on the photo‐PA and the bulk is attributed to the poor ^13^C–^13^C spin diffusion efficiency). Most importantly, the ^13^C bulk enhancement obtained with AnthraPol is 3 times larger than that observed with CarboPol. This agrees with the ratio of the average enhancements observed on the respective photo‐PAs, and demonstrates that, as expected, improvements of the local enhancements on the molecule are directly reflected in increased bulk enhancements. This trend is preserved at 14.1 T, where we find an enhancement of *ε* = –35 ± 6 for AnthraPol (Figure 3d), while it has decreased to *ε* = –4 ± 1 for CarboPol (Figure 3c). While ^13^C spin diffusion at natural abundance is inherently inefficient for application purposes, these experiments emphasize that higher local polarization levels of the photo‐PA are directly propagated to and reported by the bulk. Complementary experiments show that the ^13^C bulk enhancement for 1.5 mM AnthraPol reduces to *ε* = –19 ± 1, while the ^13^C bulk enhancement remains unchanged for CarboPol at 1.5 mM and 2.25 mM (Figures S13 and S14). We attribute this difference to a different absorption coefficient of the two photo‐PAs. While CarboPol already reached an optically saturated regime at a concentration of 1.5 mM, this is not the case for AnthraPol. This is corroborated by UV–vis experiments that suggest a one order of magnitude smaller extinction coefficient for AnthraPol compared to CarboPol (see Section S9).

**FIGURE 4:**
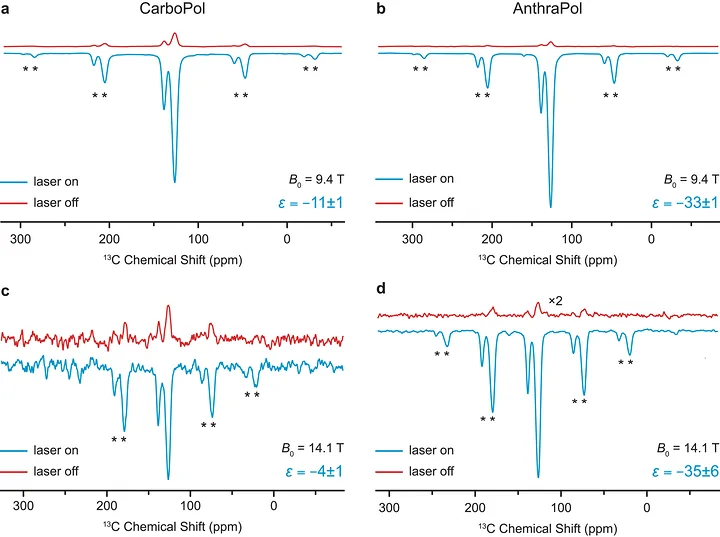
^13^C NMR spectra of a frozen solution of 1.5 mM CarboPol measured at 9.4 T (a) and 14.1 T (c) as well as 2.25 mM AnthraPol at 9.4 T (b) and 14.1 T (d) in OTP with (blue) and without (red) laser irradiation. Spectra were recorded at 100 K with a polarization delay of 5000 s under 8 kHz MAS. The asterisks denote spinning sidebands.

## 3 Conclusion

In conclusion, we have introduced a new molecular photo‐PA, AnthraPol, for carbon‐13 that yields signal enhancements up to *ε* = –4480 ± 900 corresponding to ∼16% nuclear spin polarization. Overall, the ^13^C polarization of AnthraPol per ^13^C is observed to be a factor of ∼3 larger than for CarboPol. The comparison between experimental and theoretical magnetic field dependence revealed that the efficiency of photo‐CIDNP driven by TSM might be under certain conditions dictated by factors such as Δ*ω*_e_ and not by the matching condition. Additionally, by taking advantage of ^13^C spin diffusion, we confirmed that a larger photo‐CIDNP effect on the molecule is directly translated to larger bulk enhancements. The choice for AnthraPol was based on new design principles informed by numerical simulations. Indeed, comparison with available literature data strongly suggests that factors other than the well‐known matching condition |*ω*_n_| ≈ |*d*| are the key to the improved performance of AnthraPol. Likely, a complex interplay between hyperfine coupling, kinetics of the photocycle and Δ*ω*_e_ explains the threefold better performance of AnthraPol over CarboPol. Further differentiating between these parameters will require more precise knowledge of each of these parameters. Experimental confirmation of our theoretical predictions on ^13^C photo‐CIDNP significantly advances our understanding of the spin dynamics in these molecular D–A systems, helping to guide new design principles for second‐generation photo‐PAs for dye‐sensitized NMR spectroscopy.

## Author Contributions

**Marcel Levien**: investigation, writing – original draft, methodology, validation, writing – review and editing, conceptualization. **Máté Visegrádi**: conceptualization, investigation, writing – original draft, methodology, validation, writing – review and editing. **Ganesan Karthikeyan**: investigation, methodology, writing – review and editing. **Gilles Casano**: investigation, writing – review and editing, methodology. **Christian Reiter**: investigation, methodology, writing – review and editing. **Sebastian Wegner**: investigation, methodology, writing – review and editing. **Armin Purea**: investigation, methodology, writing – review and editing. **Michael R. Wasielewski**: conceptualization, investigation, methodology, writing – review and editing, funding acquisition, validation. **Federico De Biasi**: conceptualization, methodology, investigation, writing – review and editing, writing – original draft, validation.**Olivier Ouari**: conceptualization, investigation, methodology, validation, writing – review and editing, supervision, funding acquisition. **Lyndon Emsley**: conceptualization, investigation, funding acquisition, writing – original draft, methodology, validation, writing – review and editing, project administration, supervision.

## Conflicts of Interest

The authors declare no conflicts of interest.

## Supporting information




**Supporting File 1**: The Supporting Information contains synthetic methods, experimental details, a description of the used hardware, information on the calculation of enhancements, additional NMR spectra, and complementary numerical simulations. The file also includes supporting figures and tables, and a link to all the raw NMR data.

**Supporting File 2**: anie74445‐sup‐0001‐SuppMat.pdf.

## Data Availability

The data that support the findings of this study are available from the corresponding author upon reasonable request.
