# Supplementary material for: Up to 16% Optical Carbon‐13 Hyperpolarization of Donor–Acceptor Molecules at 14.1 T
Source: Angew Chem Int Ed Engl. 2026 Sep 7;65(44):e7711870. doi: 10.1002/anie.7711870 (PMC13703767; doi:10.1002/anie.7711870)
Supplement: Supplementary file 1 [file ANIE-65-e7711870-s001.pdf]

Up to 16% Optical Carbon-13 Hyperpolarization of Donor-Acceptor Molecules at 14.1 T

Marcel Levien,[a] Máté Visegrádi,[a] Ganesan Karthikeyan,[b] Gilles Casano,[b] Christian Reiter,[c] Sebastian Wegner,[c] Armin Purea,[c] Michael R. Wasielewski,[d] Federico De Biasi,[a],[e] Olivier Ouari,[b],* and Lyndon Emsley[a],*

[a] M. Levien, M. Visegrádi, F. De Biasi, L. Emsley
Institut des Sciences et Ingenierie Chimiques, Ècole Polytechnique Fedérale de Lausanne (EPFL)
CH-1015 Lausanne, Switzerland
* E-mail: lyndon.emsley@epfl.ch

[b] G. Karthikeyan, G. Casano, O. Ouari
Aix-Marseille University, CNRS, Institut de Chimie Radicalaire
13013 Marseille, France
* Email: olivier.ouari@univ-amu.fr

[c] C. Reiter, S. Wegner, A. Purea
Bruker BioSpin GmbH & Co KG,
76275 Ettlingen, Germany

[d] M. R. Wasielewski
Department of Chemistry, Center for Molecular Quantum Transduction
Institute for Quantum Information Research and Engineering, Northwestern University
Evanston, IL 60208-3113, USA

[e] F. De Biasi
Departemnt of Chemical Sciences, University of Padove
Via Marzolo 1, 35131 Padova, Italy

WILEY-VCH

Raw NMR data

All the raw NMR data associated with the manuscript can be accessed at the following DOI: https://doi.org/10.5281/zenodo.21990543 and is available under the CC-BY-4.0 (Creative Commons Attribution-ShareAlike 4.0 International) license.

S1) Experimental details....S2

S2) Optimized light irradiation setup at 9.4 T....S3

S3) Steady-state NMR signal enhancement calculation....S5

S4) Additional NMR spectra and build up times at 9.4 T....S5

S5) ^{13}C photo-CIDNP experimental data and NMR spectra at 11.7 T, 14.1 T and 21.15 T....S7

S6) Additional numerical simulations....S10

S7) Supplementary Note 1....S10

S8) Additional bulk experiments at different photo-PA concentration....S11

S9) UV-Vis experiments..S12

S10) Synthetic procedure..S13

S11) References..S15

WILEY-VCH

S1) Experimental details

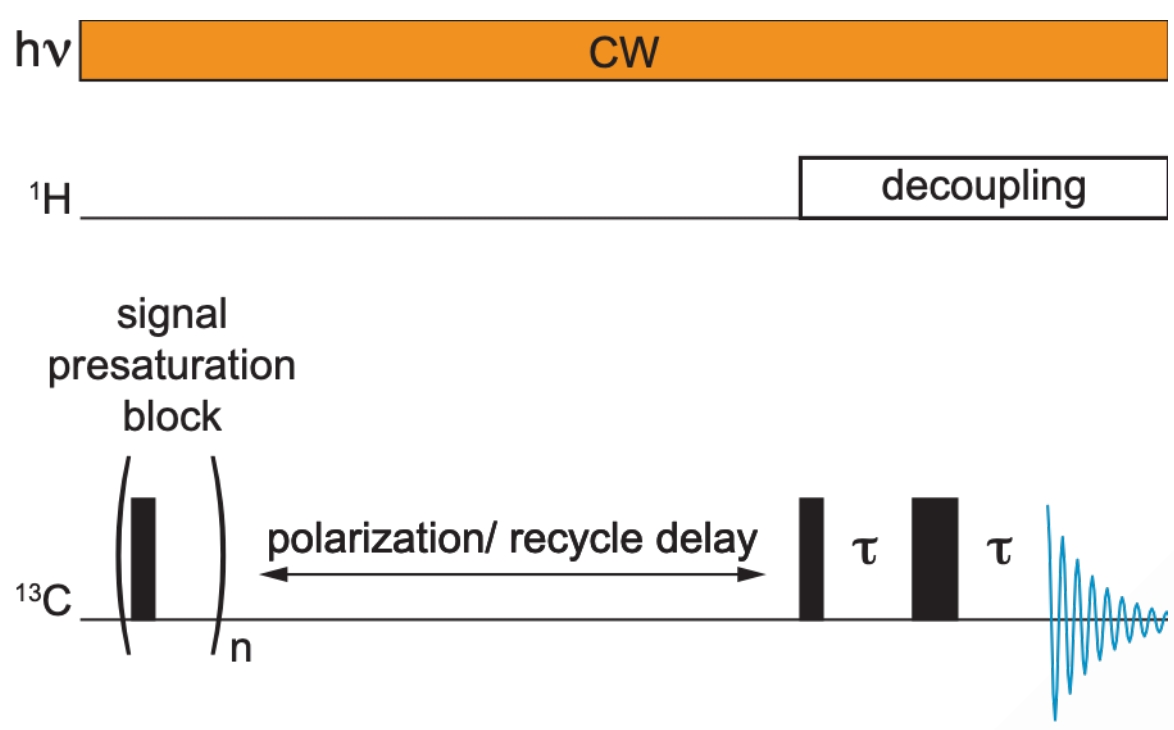

Figure S1. Spin echo pulse sequence used to collect the data in Figures 2 and 4 of the main text. After pre-saturation of the NMR signal with a series of 90° pulses, polarization builds up during the polarization delay prior to signal acquisition. The NMR signal is acquired using a rotor-synchronized Hahn echo block to suppress the probe background signal. In the experiments, *τ* has been set equal to 1 rotor periods. For measurement of build up times, the polarization delay was incremented for different delay times.

All chemicals were used as received. Samples were degassed by freeze-pump-thaw cycles prior to insertion into the magnet. Experiments were performed in 3.2 mm sapphire rotors using commercial Bruker low temperature MAS DNP probes. Experiments at all fields were performed using Bruker Avance III HD or Avance NEO spectrometer. If not noted otherwise all experiments were performed at a sample temperature of *T* ~100 K and magic angle spinning of 8 kHz. Samples were spun with a Bruker MAS II unit and as the laser irradiation interfered with the MAS reading, samples were spun in manual mode without automatic stabilization, which led to variations in MAS frequency of 8000± 20 Hz. Spectra were detected using a Hahn echo block with 1 rotor period per half echo delay. All ^{13}C experiments were acquired using ^{1}H decoupling during acquisition. If not noted otherwise, all experimental parameters were kept the same for all samples at the same magnetic field. Depending on the measurement, for experiments with 20 s recycle delay we collected 128, 256 or 512 scans, while experiments with 5000 s recycle delay were performed with 4 or 8 scans.

Table S1: NMR parameters used at different magnetic fields. The delay between saturation pulses was 1 ms. If not denoted otherwise, ^{1}H decoupling was applied to every ^{13}C detected experiment during acquisition. One saturation loop includes 20 saturation pulses. If not noted otherwise these experimental parameters were used for all shown spectra in the main text and Supplementary Information of this manuscript.

Field (T)	^{13}C 90° pulse length (µs)	^{13}C pulse power (W)	^{13}C sat. pulse length (µs)	Number of sat. loops	^{13}C sat. pulse Power (W)	^{1}H decoupling sequence	^{1}H dec. pulse length (µs)	^{1}H dec. pulse power (W)
9.4	4	70	1000	100	2	Spinal64	5.1	80
11.7	5	86	1000	50	2	Spinal64	6.61	60
14.1	2.5	60	1000	1	2	Spinal64	5.8	70
21.1	3.3	138	1000	200	2	Spinal64	5.8	80

S2) Optimized light irradiation setup at 9.4 T

a b

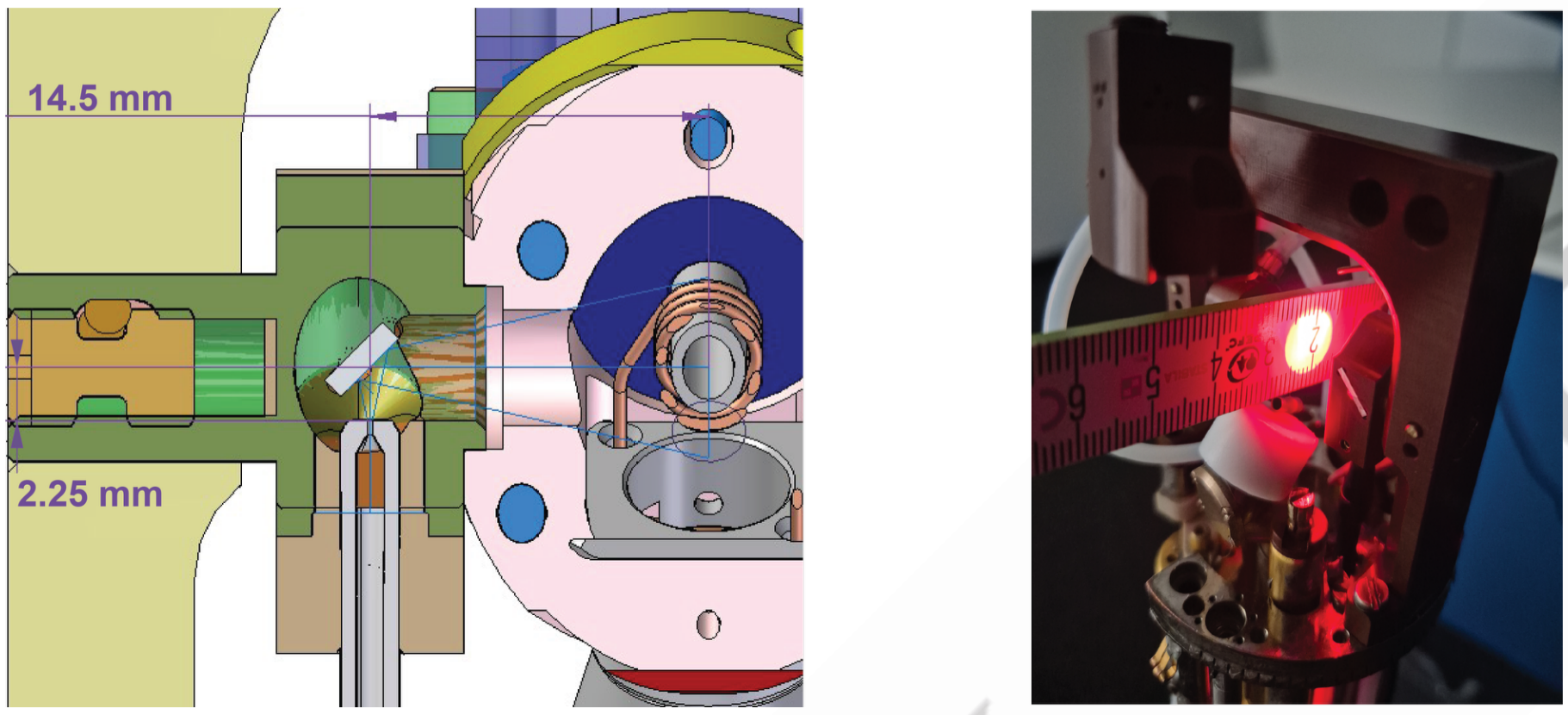

Figure S2. (a) Drawing of the top of the probe at 9.4 T. The optical fiber (grey) is fed from the bottom into the variable temperature gas flow channel. A mirror directs the beam onto the sample. (b) Photo of the light beam in the sample area of the modified probe.

As a light source, we used 450 nm blue laser, which is composed of a diode with 3 W nominal output power controlled by an ACAN A12 driver and an adjustable focal length. The beam is focused in a collimator using one or two mirrors, which then feed the laser beam into an optical fiber via SMA connector.

Our previous approach of light delivery to the sample[1] was achieved by feeding an optical fiber through the µwave waveguide and place the bare end of the fiber as close as possible to the sample. This enabled irradiation of the sample, however modifications of the probe were necessary before each experimental session. Slight misalignment of the optical fiber could lead to reduced signal enhancements and thus cause delays in the experimental workflow. To eliminate these problems and simplify the experimental setup, we modified our low temperature MAS DNP probe to permanently accommodate an optical fiber that enables efficient light irradiation of the sample area at low temperature. On the bottom of the probe an SMA feedthrough connects the internal fiber with an external fiber that delivers the light beam from the source. To irradiate the sample, the fiber is fed from the bottom into the variable temperature gas channel. At the end of the fiber the light is emitted onto a mirror that directs the beam to the sample ensuring efficient irradiation of the rotor (Figure S1). To assess the performance of these two approaches, we compared ^{1}H and ^{13}C photo-CIDNP enhancements obtained with three different photo-PAs. The results are depicted in Figure S2 and show very similar performance of the two approaches. Experiments at magnetic fields other than 9.4 T were performed using the old approach by feeding an optical fiber through a µwave waveguide or an additional opening on the bottom of the probe and place the bare end of the fiber as close as possible to the sample.

WILEY-VCH

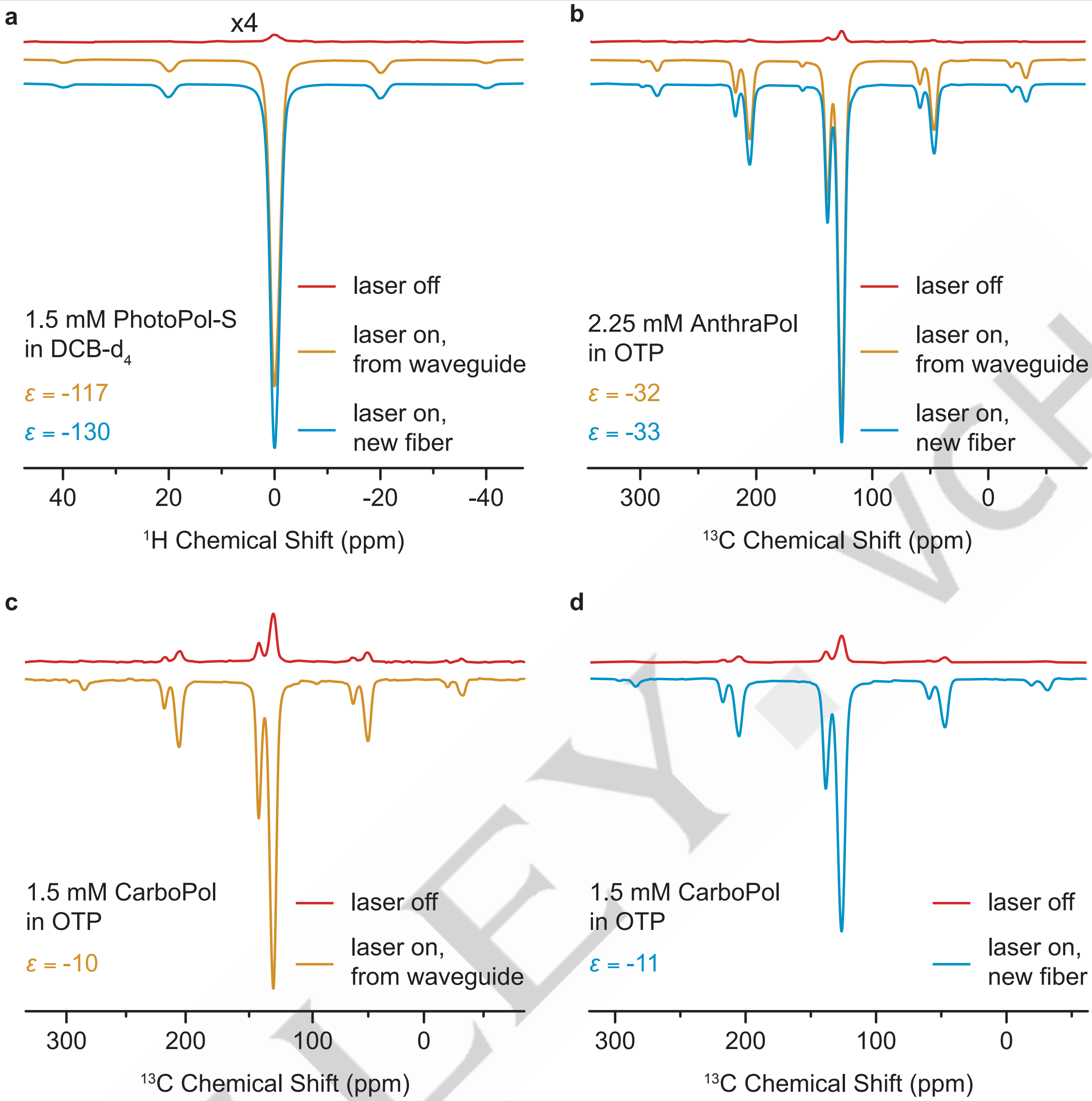

Figure S3. (a) ^{1}H NMR spectra of a frozen solution of dichlorobenzene-d_4 (DCB-d_4) using 1.5 mM PhotoPol-S as the photo-PA. We used a relaxation delay (RD) of 20 s and 8 scans per spectrum. For a 90° pulse at 2.5 μs, we used 60 W. (b) ^{13}C NMR spectra of a frozen solution of 2.25 mM AnthraPol in OTP. We used a relaxation delay of 5000 s and 4 scans per spectrum. (c) and (d) show the ^{13}C NMR spectra of a frozen solution of 1.5 mM CarboPol in OTP acquired in different experimental sessions. Spectra in (c) and (d) were collected with RD = 5000 s and 8 and 4 scans for the laser off (red) and laser on (yellow and blue) experiments, respectively. All experiments were performed at 9.4 T, 100 K, 8 kHz MAS, using a Hahn echo delay pulse sequence with 4 (^{1}H) or 1(^{13}C) rotor periods per half echo delay, Red spectra were measured without laser irradiation, while the yellow and blue spectra were obtained under light illumination using an optical fiber inserted through the waveguide and the newly installed permanent fiber, respectively.

WILEY-VCH

S3) Steady-state NMR signal enhancement calculations

In order to obtain the steady-state signal enhancements of hyperpolarized carbon sites of the photo-PA, we took advantage of the different build up times of the photo-PA carbons and the ^{13}C signals arising from the solvent OTP. Indeed, as shown in Figures S3-S4, the build up time of the ^{13}C nuclei on the donor-acceptor molecules is ~3 – 25 s, while it is ≥ 1000 s for the ^{13}C nuclei of the solvent OTP. Additionally, the carbon concentration of OTP is ~91 M, while we used at most 2.25 mM of photo-PA. Therefore, we can safely assume that at sufficiently long RD and without laser irradiation, the observed signals originate exclusively from OTP carbons. The enhancement of the respective carbon sites can then be calculated by comparing the integrated signal intensity of the single peaks (including their sidebands) under light illumination at RD = 20 s to the integral over the whole spectrum obtained at an RD = 12500 s without laser irradiation. Scaling by the relative ^{13}C concentration and number of scans yields the steady-state enhancement according to:

$$\varepsilon \approx \frac{I_{\text{carbon site}}(\text{RD} = 20\text{ s, laser on})}{I_{\text{whole spectrum}}(\text{RD} = 12500\text{ s, laser off})} \times \frac{NS(\text{RD} = 12500\text{ s, laser off})}{NS(\text{RD} = 20\text{ s, laser on})} \times \frac{c(\text{carbons in OTP})}{c\ (\text{carbon site in photo} - \text{PA})},$$

where *I* is the respective integral of the signal, NS means number of scans, and *c* is the concentration. We note that for integration all side bands of the same peak need to be considered.

S4) Additional NMR spectra and build up times at 9.4 T

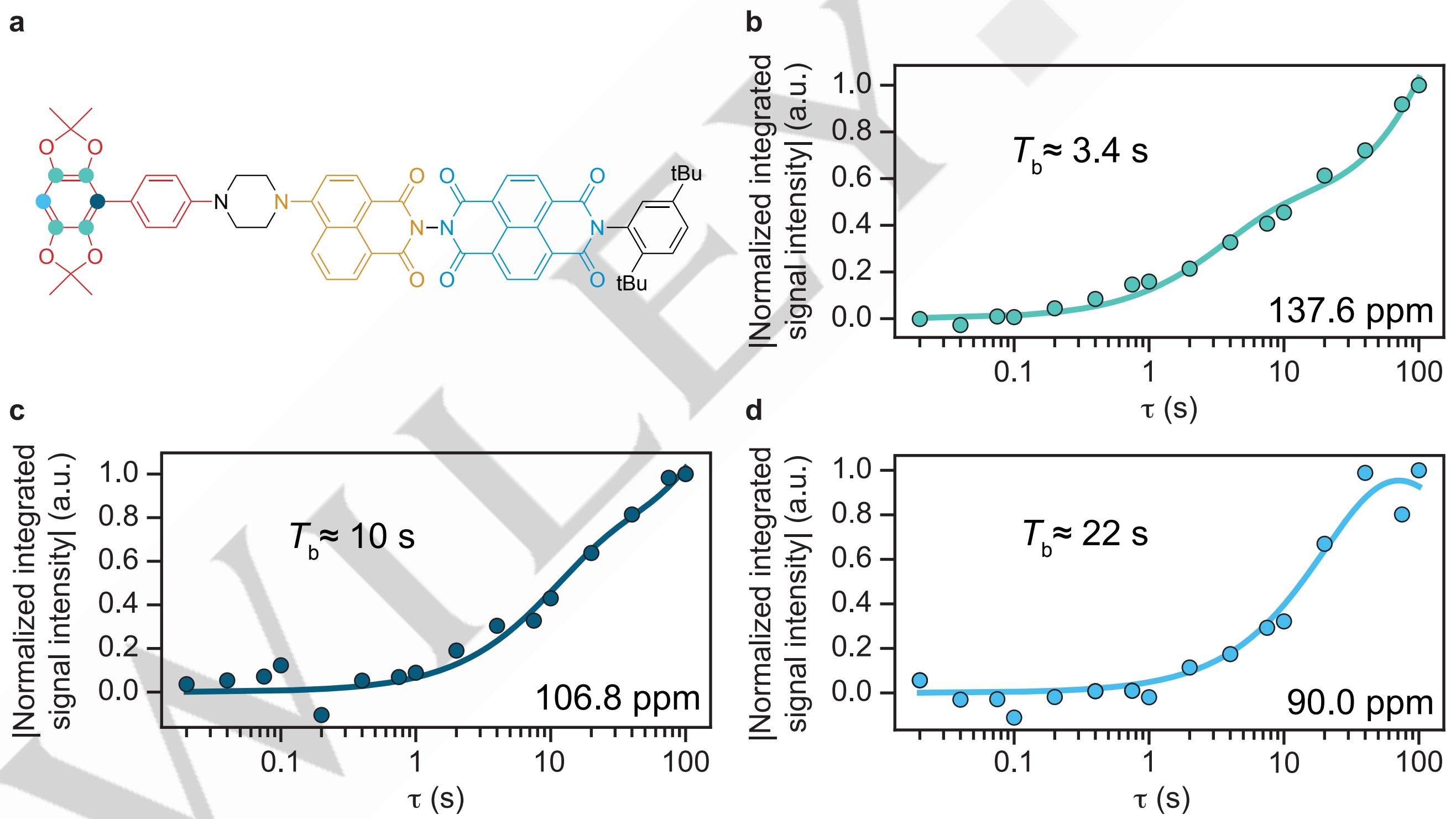

Figure S4. (a) Structure of CarboPol. T_b was obtained for the highlighted carbon sites. (b-d) ^{13}C NMR polarization build up curves at 100 K under light illumination for the respective carbon sites. The color code is given in (a). The solid line is a best fit to a biexponential function $I(\tau) = I_{\text{fast}}\left(1 - \exp\left(\frac{-\tau}{T_{\text{b,fast}}}\right)\right) + I_{\text{slow}}\left(1 - \exp\left(\frac{-\tau}{T_{\text{b,slow}}}\right)\right)$, where the fast time constant $T_{\text{b,fast}}$ corresponds to the build up times of the different ^{13}C carbon sites of CarboPol, while $T_{\text{b,slow}}$ is assigned to the build up time of the OTP solvent ^{13}C nuclei and was in all cases ≥ 1000 s. We recently reported a build up time of $T_{\text{b,fast}}$ = 1.8 s for the peak at 137.6 ppm.[2] Considering the uncertainty of the fit, our data is in agreement with our previous report.

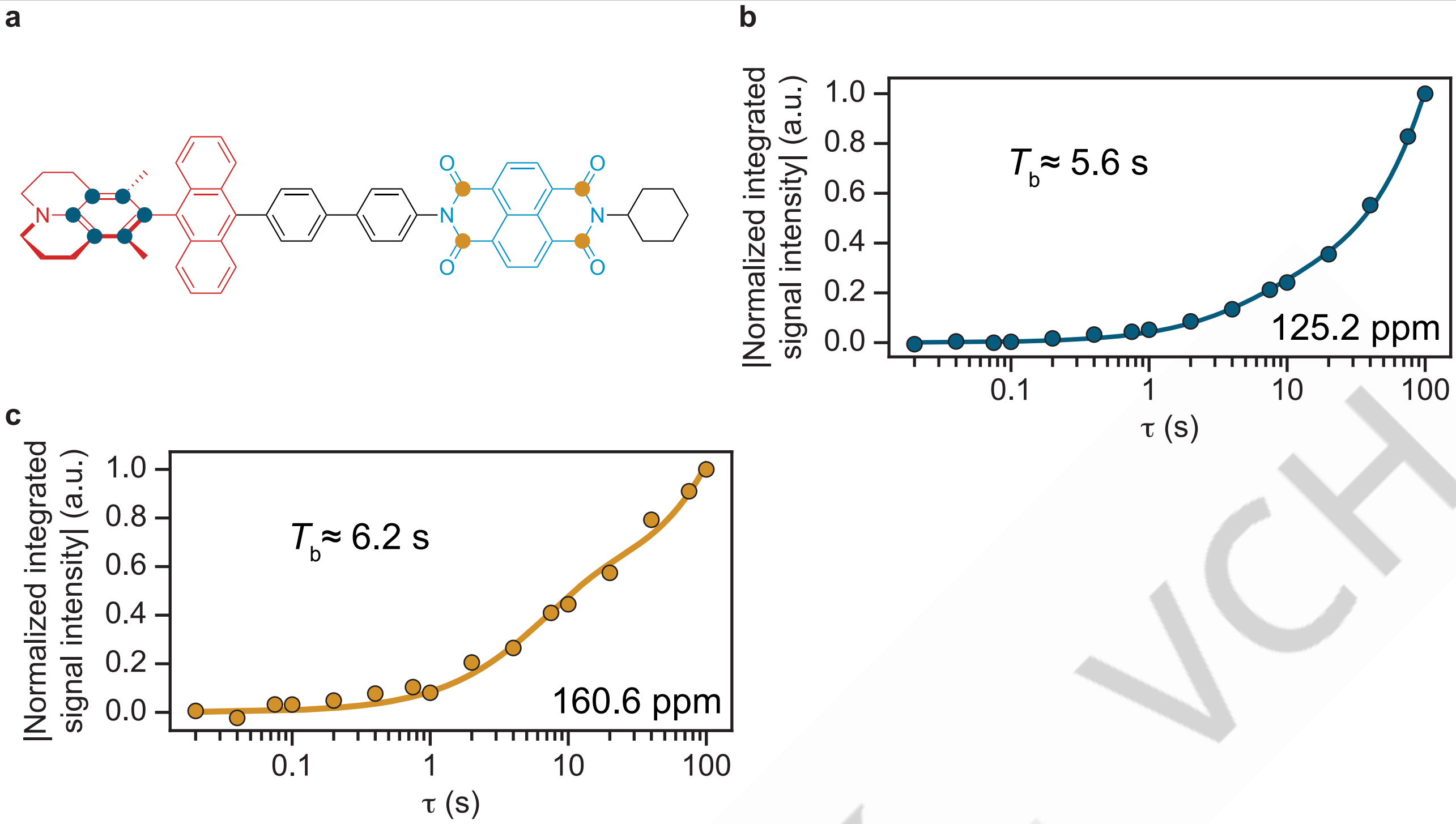

Figure S5. (a) Structure of AnthraPol. T_b was obtained for the highlighted carbon sites. (b-c) ^{13}C NMR polarization build up curves at 100 K under light illumination for the respective carbon sites. The color code is given in (a). The solid line is a best fit to a biexponential function $I(\tau) = I_{\mathrm{fast}}\left(1 - \exp\left(\frac{-\tau}{T_{\mathrm{b,fast}}}\right)\right) + I_{\mathrm{slow}}\left(1 - \exp\left(\frac{-\tau}{T_{\mathrm{b,slow}}}\right)\right)$, where the fast time constant $T_{b,fast}$ corresponds to the build up times of the different ^{13}C carbon sites of CarboPol, while $T_{b,slow}$ is assigned to the build up time of the OTP solvent ^{13}C nuclei and was in all cases ≥ 1000 s.

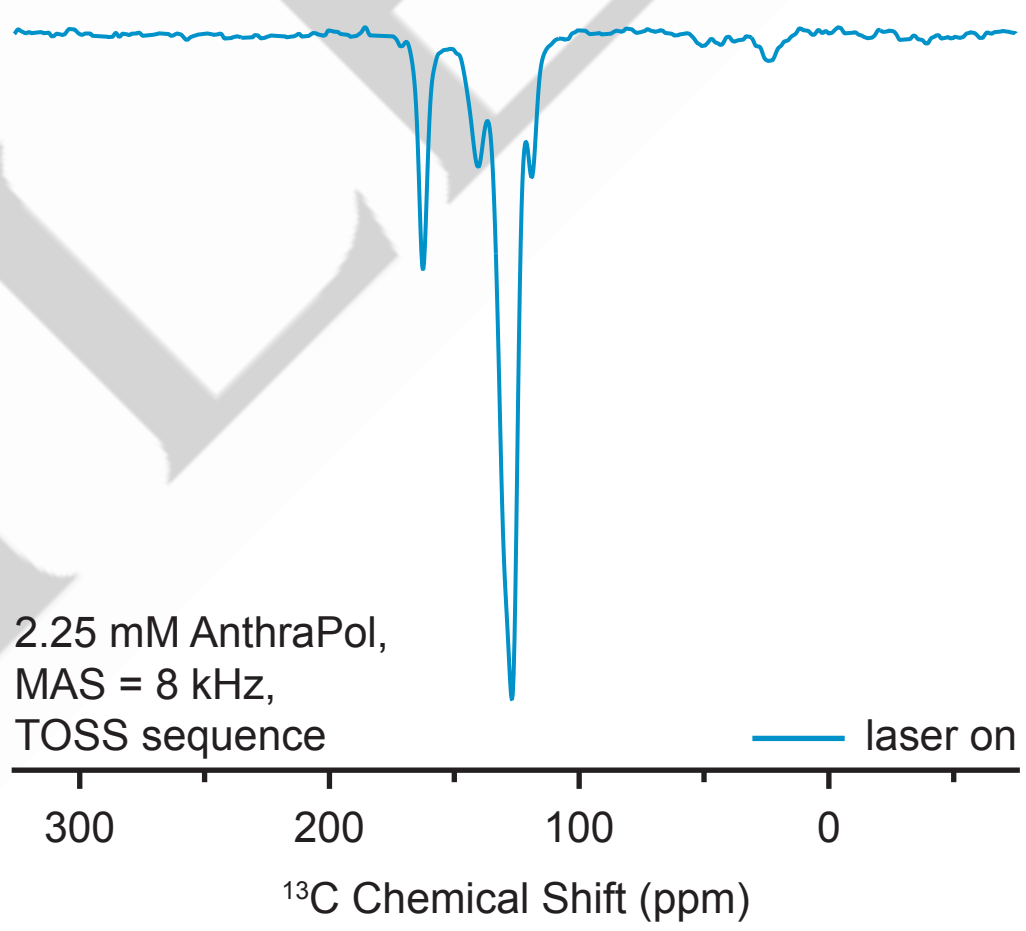

Figure S6. ^{13}C NMR spectrum using the TOSS pulse sequence und light illumination, MAS = 8 kHz and 100 K. 90° pulse length was 2.5 µs @ 96.2 W We modified the standard Bruker cptoss sequence to be compatible with direct ^{13}C detection. The TOSS sequence was employed using 2.24 rotor evolutions. The spectrum was acquired with 32 scans and RD = 20 s and clearly shows that there are not signals hidden below the sidebands arising from the main peak.

WILEY-VCH

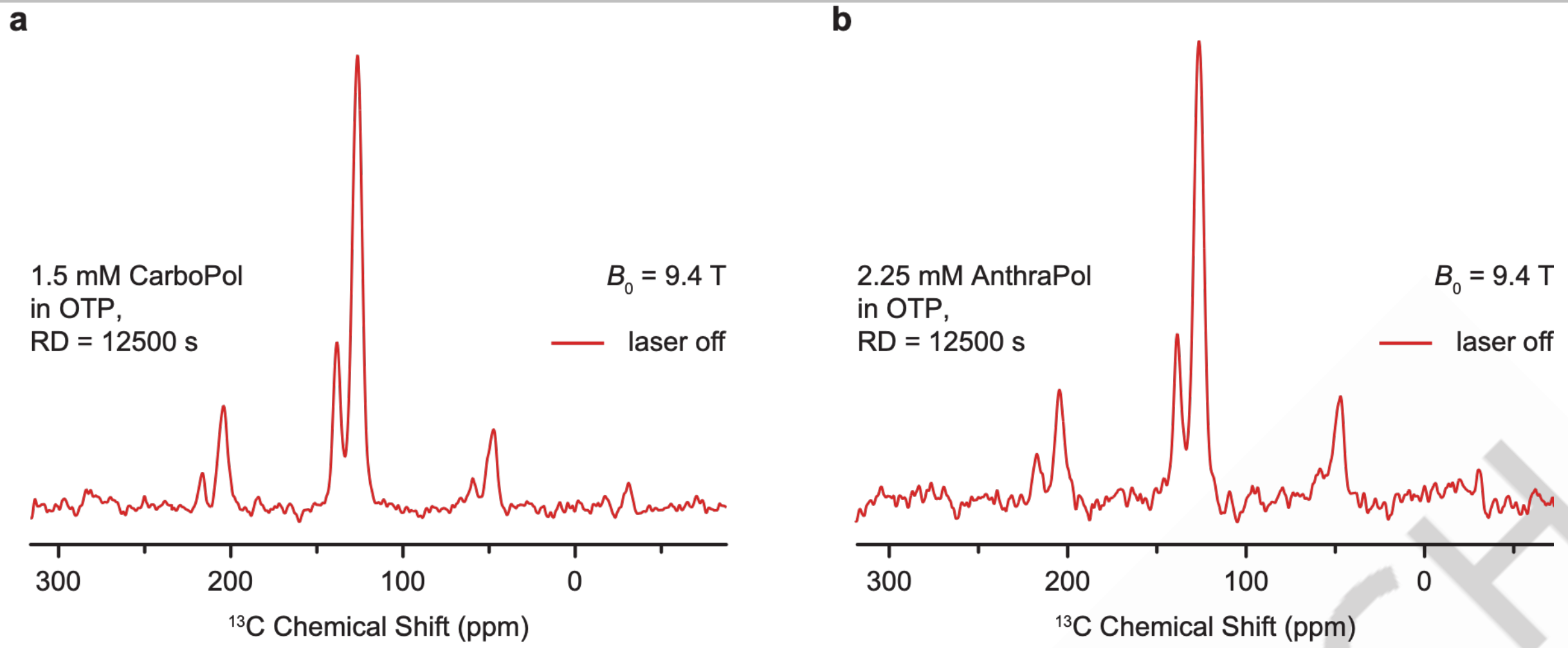

Figure S7. ^{13}C NMR spectra of a frozen solution of 1.5 mM CarboPol (a) and 2.25 mM AnthraPol (b) in OTP without light illumination. We used a relaxation delay of 12500 s and 4 scans per spectrum. All experiments were performed at 9.4 T, 100 K, 8 kHz MAS, using a Hahn echo delay pulse sequence with 1 rotor period per half echo delay.

S5) ^{13}C photo-CIDNP experimental data and NMR spectra at 11.7 T, 14.1 T and 21.15 T

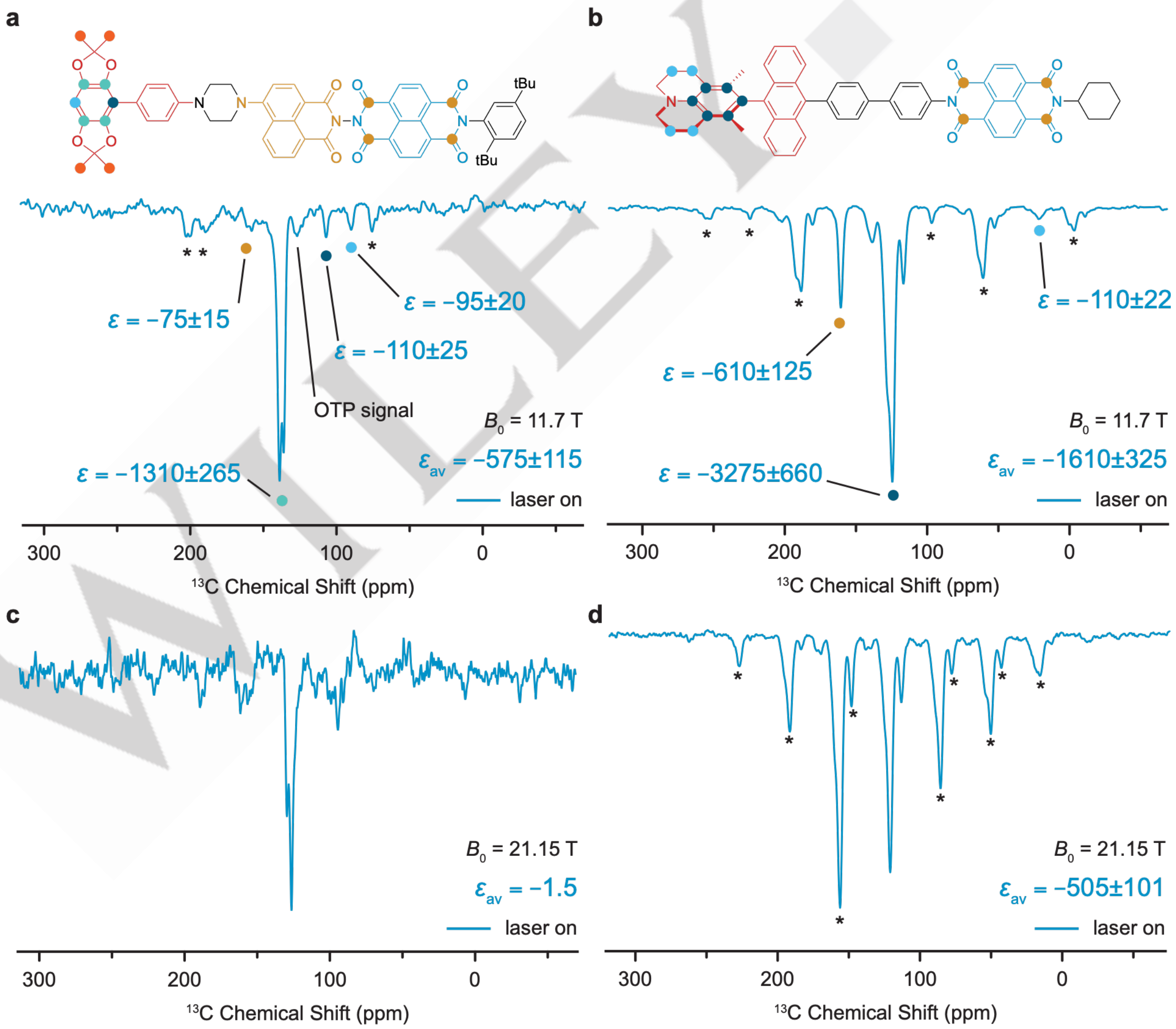

Figure S8. ^{13}C NMR spectrum of a 1.5 mM frozen solution of CarboPol in OTP under continuous light irradiation (450 nm, ~1.2 W output laser power), at 11.7 T, with a sample temperature of 100 K and with 8 kHz MAS. The polarization delay between scans was set to 20 s to ensure that almost no OTP signal was observed. The colored dots on the resonances indicate the assignment to the different ^{13}C nuclei indicated on the chemical structure of CarboPol shown above the spectrum. (b) ^{13}C NMR spectrum of a 2.25 mM frozen solution of AnthraPol in OTP under identical experimental conditions as in (a). (c) ^{13}C NMR spectrum of a 1.5 mM frozen solution of CarboPol in OTP at 21.15 T. All other parameters were identical to (a). (d) ^{13}C NMR spectrum of a 2.25 mM frozen solution of AnthraPol in OTP at 21.15 T. All other parameters were identical to (b). Strong chemical shift anisotropy prevented specific assignment of the spectra collected at 21 T. Instead only the average enhancement is reported assuming the same carbons contribute to the spectra as at lower magnetic field.

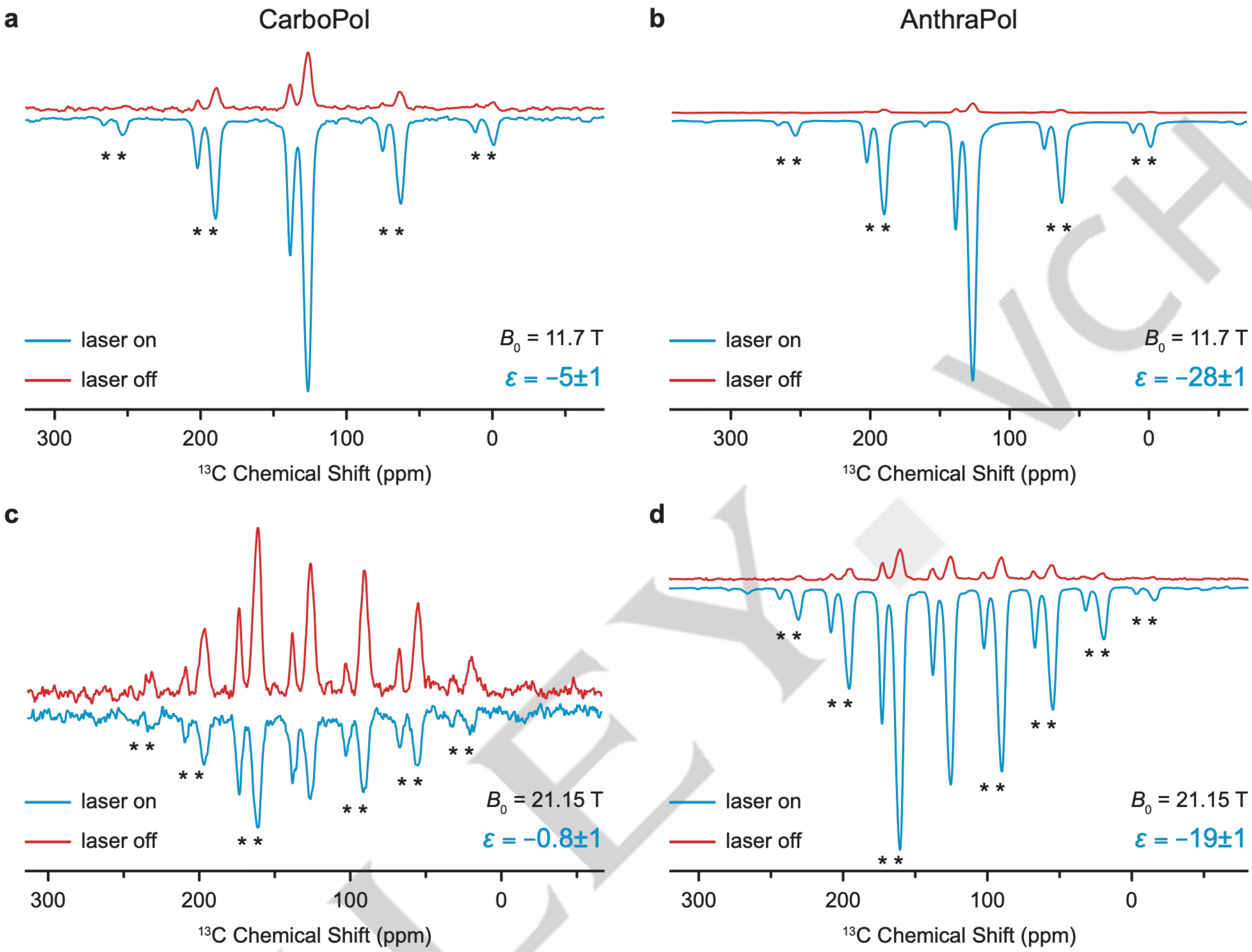

Figure S9. ^{13}C NMR spectra of a frozen solution of 1.5 mM CarboPol measured at 11.7 T (a) and 21.15 T (c) as well as 2.25 mM AnthraPol at 11.7 T (b) and 21.15 T (d) in OTP with (blue) and without (red) laser irradiation. Spectra were recorded at 100 K with a polarization delay of 5000 s under 8 kHz MAS. Acquisition of the spectra shown in d was achieved using a 90° pulse length was 2.5 μs @ 282 W. All other experiments were acquired with the parameters detailed in table S1. The asterisks denote spinning sidebands.

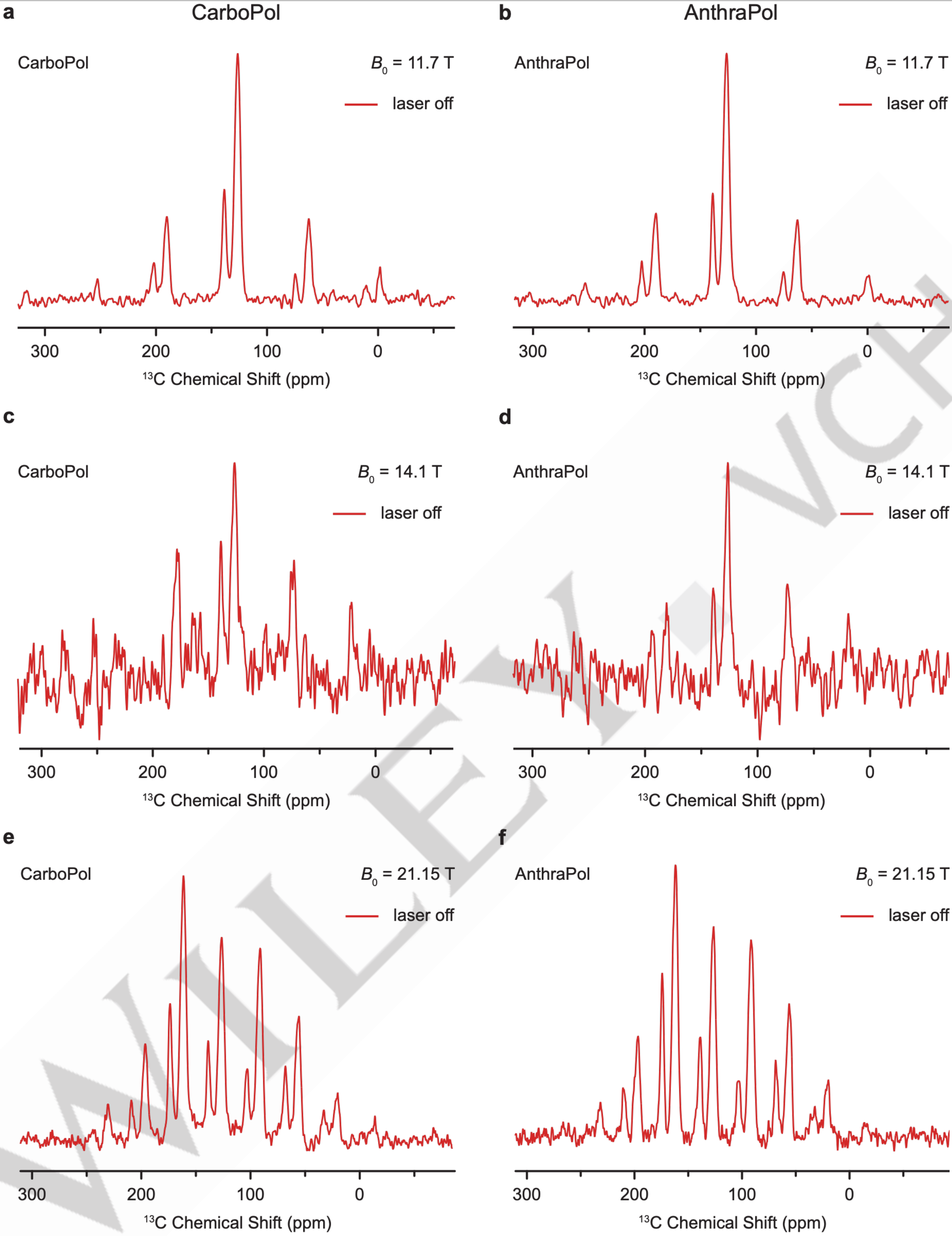

Figure S10. ^{13}C NMR spectra of a frozen solution of 1.5 mM CarboPol (a,c,e) and 2.25 mM AnthraPol (b,d,f) in OTP without light illumination. We used a relaxation delay of 12500 s and 4 scans per spectrum. Experiments were performed at 11.7 T (a,b), 14.1 T (c,d) and 21.15 T (e,f). All experiments were performed at 100 K, 8 kHz MAS and using a Hahn echo delay pulse sequence with 1 rotor period per half echo delay.

S6) Additional kinetic photocycle calculations

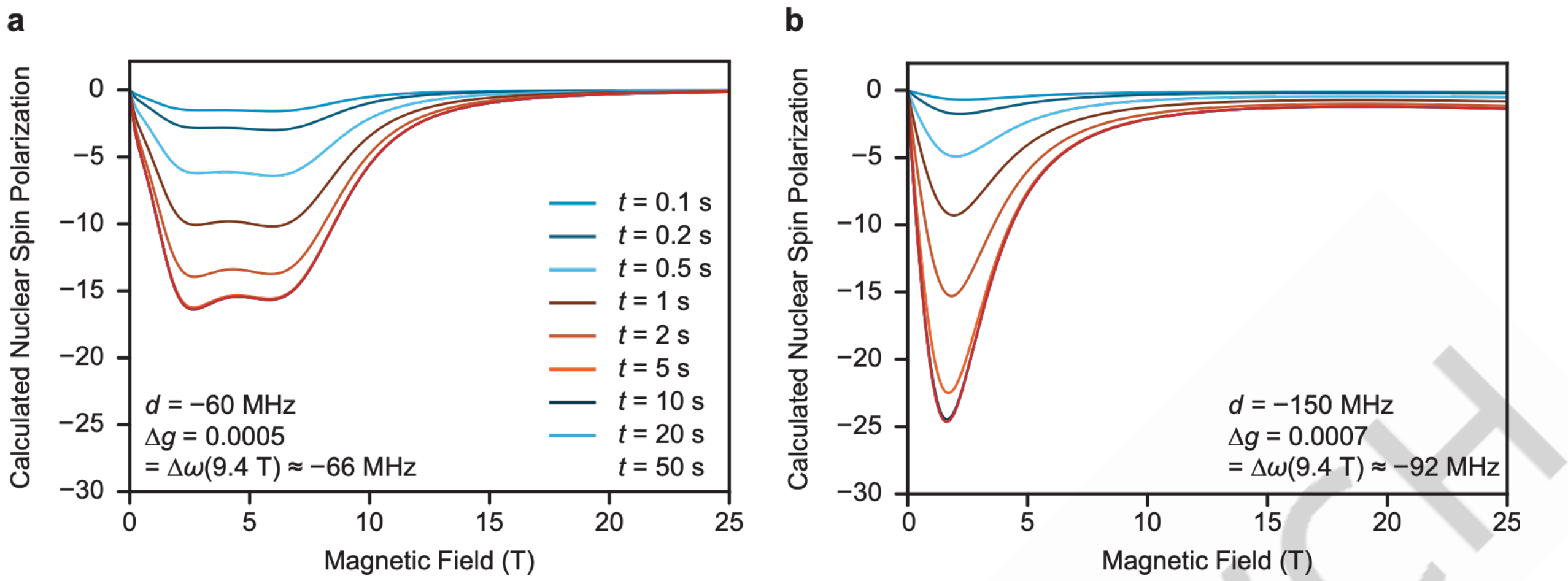

Figure S11. (a) and (b) Calculated nuclear spin polarization as a function of the magnetic field using our recently introduced kinetic photocycle treatment based on the Liouville von Neumann equation $\frac{d}{dt}\tilde{\rho} = i\tilde{H}\tilde{\rho} - \tilde{R}\tilde{\rho}$ and $\tilde{\rho}(t) = \exp[(-i\tilde{H} + \tilde{R})\tilde{\rho}_0]$.[3] We used the same parameters in (c) and (d) with the exception of d and Δg ($\Delta\omega_e$), which are given in the insets. Other parameters were chosen according to the example data set used in our recent study: k_{dec} = 10^1 s^{-1}, k_{exc} = 10^2 s^{-1}, k_s = 2.22 × 10^7, k_T = 2.0 × 10^8 s^{-1}, T_{1gs} = 5 s, T_{1nT} = 1 s, a = 20 MHz and b = 5 MHz.[3]

S7) Supplementary Note 1

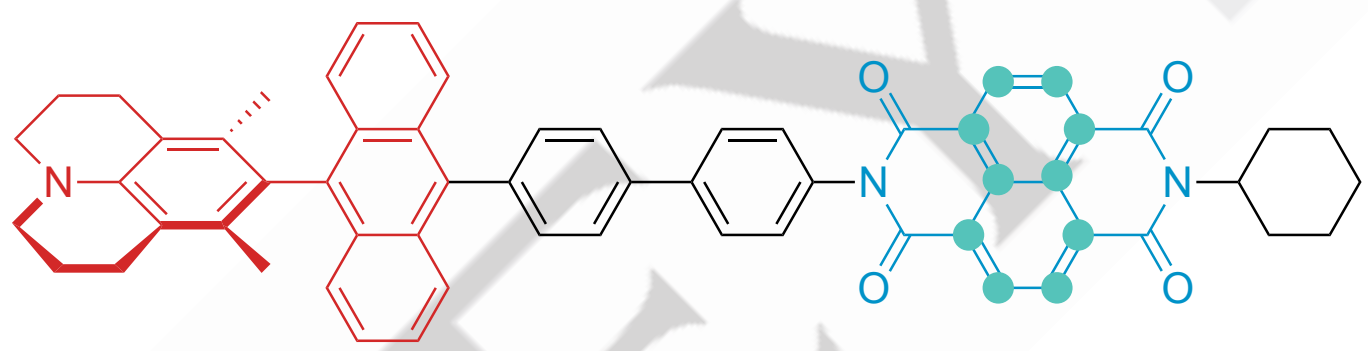

Figure S12. Structure of AnthraPol. Here, the ten aromatic carbon sites of the NDI are highlighted.

Earlier assignment of CarboPol[2] suggests that the chemical shift of the ten aromatic carbons of the NDI (see turquoise circles in Supplementary Figure S12) likely overlap with the main peak and with OTP. A spectrum collected at short recycle delay (1.8. s) shows only a slight reduction in OTP signal that could potential be caused by the underlying aromatic NDI carbons. Note, at such short recycle delay, the signal of OTP remains positive and is not inverted by spin diffusion of hyperpolarization from the photo-PA. By comparison to other features in the spectrum, this reduction is somewhat comparable to the methylene signal (scaled by the different amount of carbons contributing to the resonance), which would correspond to an approximate enhancement of ε = 45± 9.

For AnthraPol, the hyperpolarization of the NDI carbons could be more efficient as demonstrated by a seven-fold enhancement increase for the NDI carbonyl carbons, however this highly depends on the hyperfine coupling of each carbon as well as on the PRE effect. Assuming also a seven-fold change in enhancement, the resonance of these ten carbons could be enhanced by ε ≈ 45 × 2.5 × 7 ≈ 788. The enhancement of a fully resolved main peak could therefore show a reduction of enhancement by the same amount. This reduction is already included in the error bar of 20 % that were used to calculate the experimental uncertainty.

S8) Additional bulk experiments at different photo-PA concentration

Due to availability, comparative CarboPol experiments were performed using a slightly modified version of CarboPol termed CarboPol-cy-d12. To verify that the performance is similar to CarboPol, we report the bulk enhancement of CarboPol-cy-d12 at 1.5 mM in OTP in Figure S13. The bulk enhancement, i.e. laser on/off, at a recycle delay of 5000 s of 1.5 mM AnthraPol in OTP and 2.25 mM CarboPol-cy-d12 in OTP is shown in Figure S14.

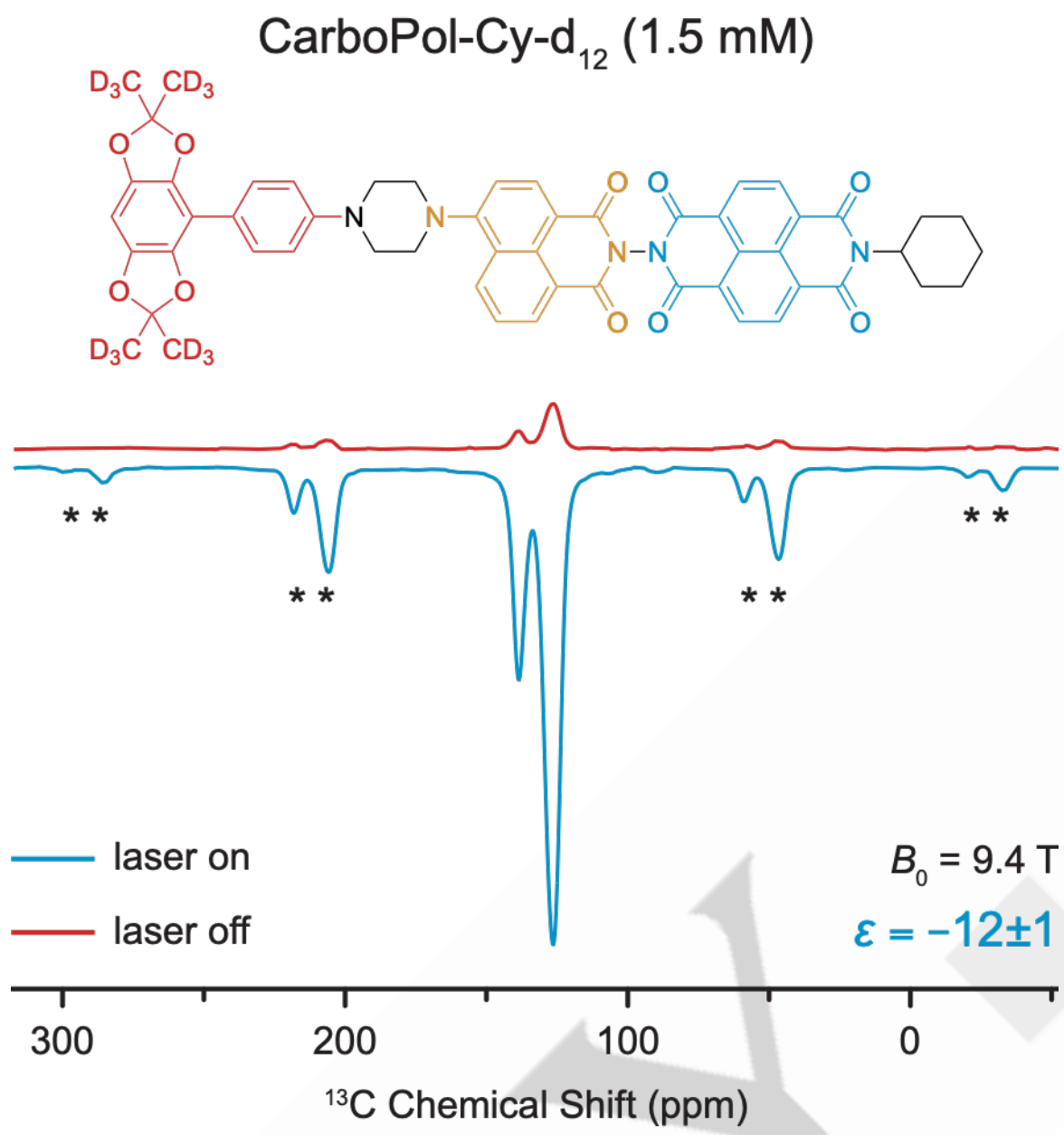

Figure S13. Upper: Structure of CarboPol-Cy-d12. ^{13}C NMR spectra of a frozen solution of 1.5 mM CarboPol-Cy-d12 measured at 9.4 in OTP with (blue) and without (red) laser irradiation. Spectra were recorded at 100 K with a polarization delay of 5000 s under 8 kHz MAS. The asterisks denote spinning sidebands.

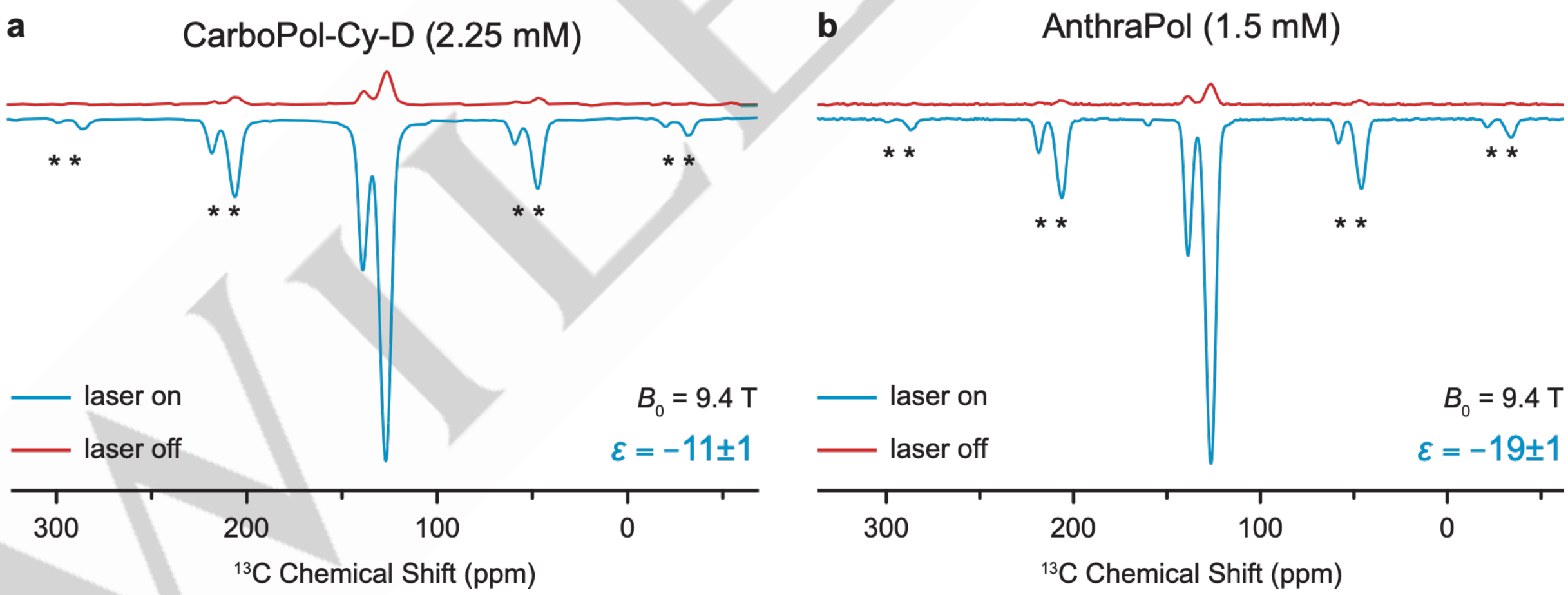

Figure S14: ^{13}C NMR spectra of a frozen solution of 2.25 mM CarboPol-Cy-d12 measured and 1.5 mM AnthraPol at 9.4 T in OTP with (blue) and without (red) laser irradiation. Spectra were recorded at 100 K with a polarization delay of 5000 s under 8 kHz MAS. The asterisks denote spinning sidebands.

S9) UV-Vis experiments

To confirm the lower absorption of AnthraPol compared to CarboPol, we performed liquid-state room temperature UV-Vis experiments as a function of the photo-PA concentration. To 2 mL of neat toluene (no degassing was applied), we step wise added 5 µL of a 1.5 mM of CarboPol and AnthraPol, respectively. CarboPol was dissolved in $CDCl_3$, while the solvent for AnthraPol was 1,2-Dichlorobenzene (DCB). We verified that the small amounts of $CDCl_3$/DCB had no impact on the UV-Vis spectrum by comparing a spectrum of neat toluene to a spectrum, where we added either 40 µL of $CDCl_3$ or 60 µL DCB to toluene, while not adding any photo-PA. We used a quartz cuvette with l = 1 cm. Experiments were performed on an Agilent Cary 60 UV-Vis. We measured between 300 and 500 nm in dual beam mode using an averaging time of 0.5 s at a scan rate of 600 nm/min and in 5 nm steps. We collected triplicates of each data point and used the mean average. As we observed a slight baseline offset, we subtracted the value at 500 nm from each data point. We also collected the UV-Vis spectrum of neat toluene and subtracted this (after baseline correction) from each of the acquired spectra.

Figure S15 shows a representative UV-Vis spectrum of CarboPol and AnthraPol, which was used to obtain the Lambert-Beer plots at 450 nm. The extracted extinction coefficients ε for CarboPol and AnthraPol are 2480 and 243 L · mol^{-1} · cm^{-1}, respectively. Therefore, the absorption of CarboPol is about one order of magnitude better than for AnthraPol. This likely explains the different bulk performance of CarboPol and AnthraPol. The data are also in agreement with previous published data that indicates that the main absorption band for the chromophore of CarboPol is around 415 – 420 nm,[4] while the one of the AnthraPol chromophore is about 380 – 400 nm[5] and therefore even farther away from the irradiation frequency of 450 nm. We note that the irradiation frequency is chosen to selectively excite the chromophore.

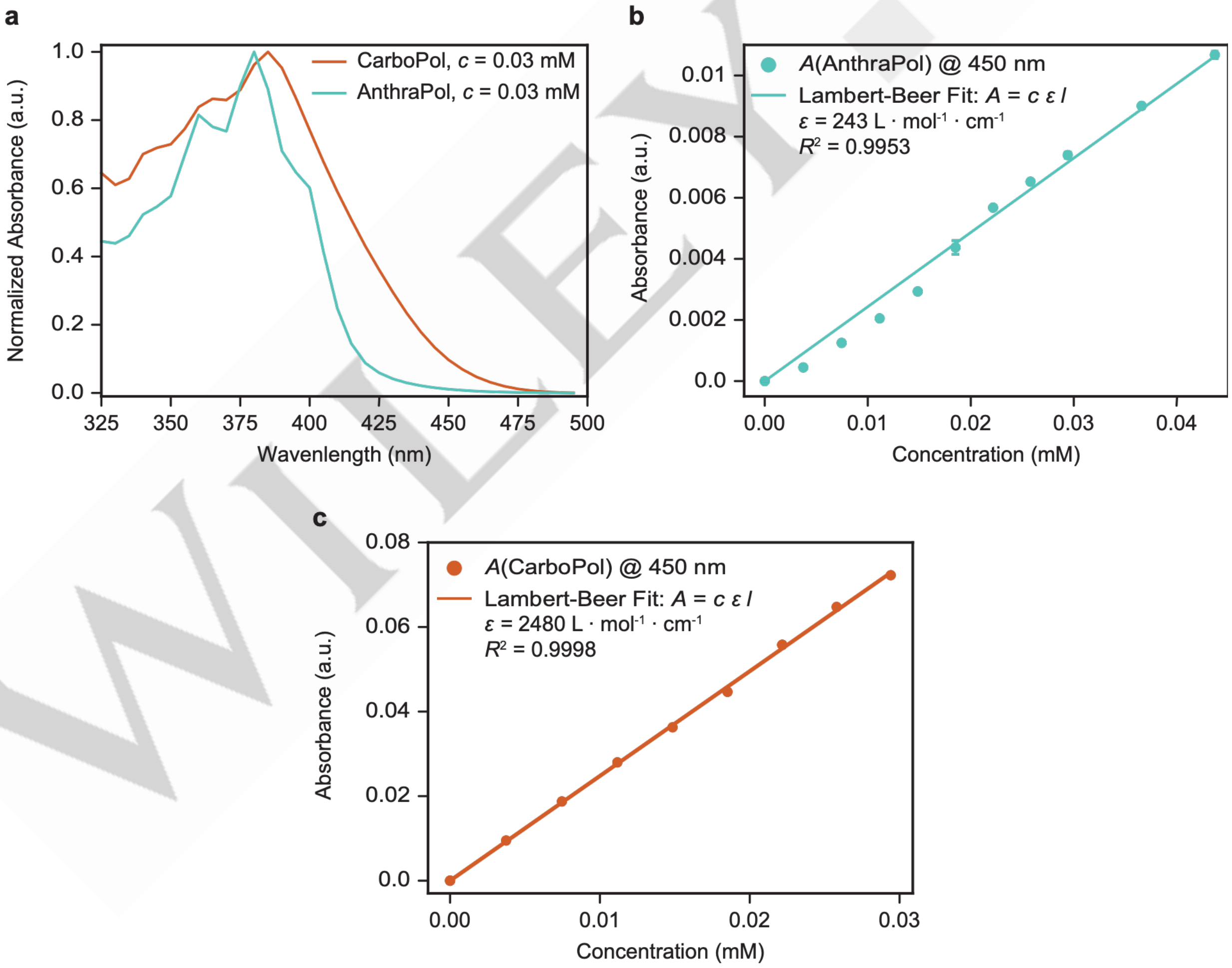

Figure S15: (a) Liquid-state room temperature UV-VIS spectrum of CarboPol (orange) and AnthraPol (turquoise). Experiments were performed in toluene. (b) Lambert-Beer plot of AnthraPol at a wavelength of 450 nm, which is the nominal wavelength of the laser used in the solid-state photo-CIDNP experiments. Turquoise circles are experimental data and the solid line is a linear fit. (c) The same Lambert-Beer plot as in (b) but using CarboPol as the photo-PA. The length of the quartz cuvette is 1 cm. If not displayed, error bars are within the size of the symbol.

S10) Synthetic procedures

General procedures: Chemicals were used as received from the suppliers. Reactions were monitored by thin-layer chromatography (TLC) analysis. Column chromatography was carried out on silica gel (230-400 mesh). The ^{1}H NMR spectra were recorded on a Bruker AVL spectrometer at 400 MHz. The chemical shifts are reported in ppm downfield relative to TMS and referenced using the residual CHCl3 resonance (δ = 7.26) for ^{1}H NMR. ESI-HRMS was performed on a SYNAPT G2 HDMS (Waters). The NMR and MS analysis were performed at the Spectropole facilities, Marseille.
Compound A was synthesized from 3,5-dimethylaniline in 6 steps by following reported procedure.[6] Compound B was prepared in 1 step as previously reported.[7]

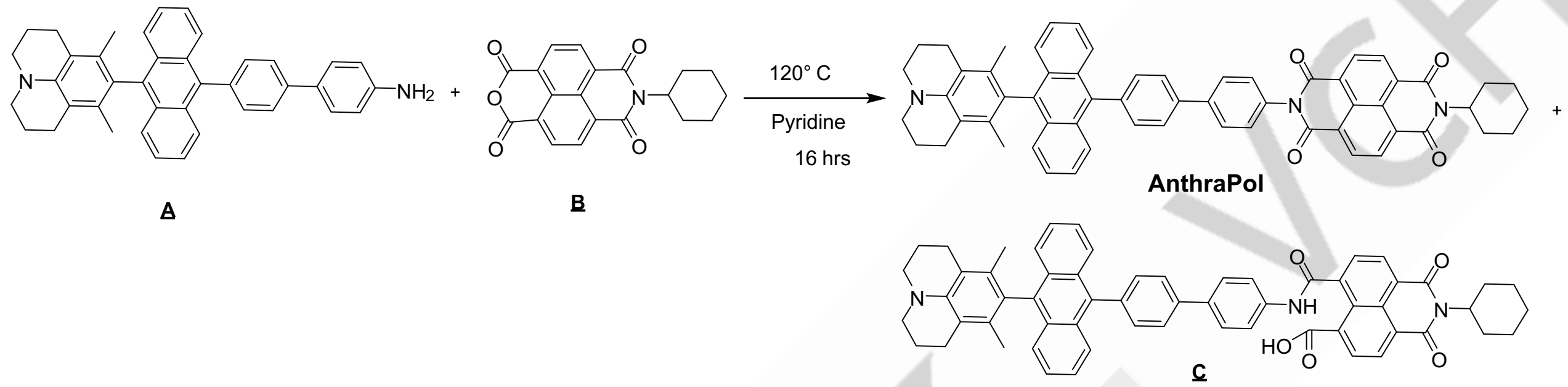

Compound **A** (20 mg, 0.037 mmol) and *N*-cyclohexyl-1,4,5,8-naphthalene- tetracarboxymonoimide (**B**, 12 mg, 0.034 mmol) were dissolved in 3mL of pyridine and the mixture was heated at 120 °C under an argon atmosphere for 16 hours. The reaction mixture was concentrated in vacuum and purified by column chromatography to yield **AnthraPol** (6mg, 20%), R_f = 0.5 (100 % $CHCl_3$) and uncyclized intermediate **C** (8mg, confirmed by LCMS).

^{1}H NMR ($CDCl_3$, 400 MHz) *δ*= 8.87-8.80 (m, 4H), 7.98 (d, *J*=8.0 Hz, 2H), 7.90 (d, *J*=8.0 Hz, 2H), 7.80 (d, *J*=8.0 Hz, 2H), 7.67-7.62 (m, 4H), 7.50 (d, *J*=8.4 Hz, 2H), 7.37-7.33 (m, 4H), 5.09-5.02 (m, 1H), 3.21 (t, *J*=5.2 Hz, 4H), 2.76 (t, *J*=6.8 Hz, 4H), 2.61-2.52 (m, 2H), 2.16-2.13 (m, 4H), 1.97-1.93 (m, 2H), 1.80-1.75 (m, 3H), 1.60 (s, 6H), 1.38-1.32 (m, 3H).

HRMS (ESI-TOF) m/z: $[M+H]^+$ calcd for $C_{60}H_{50}N_3O_4^+$ 876.3796, found 876.3778.

WILEY-VCH

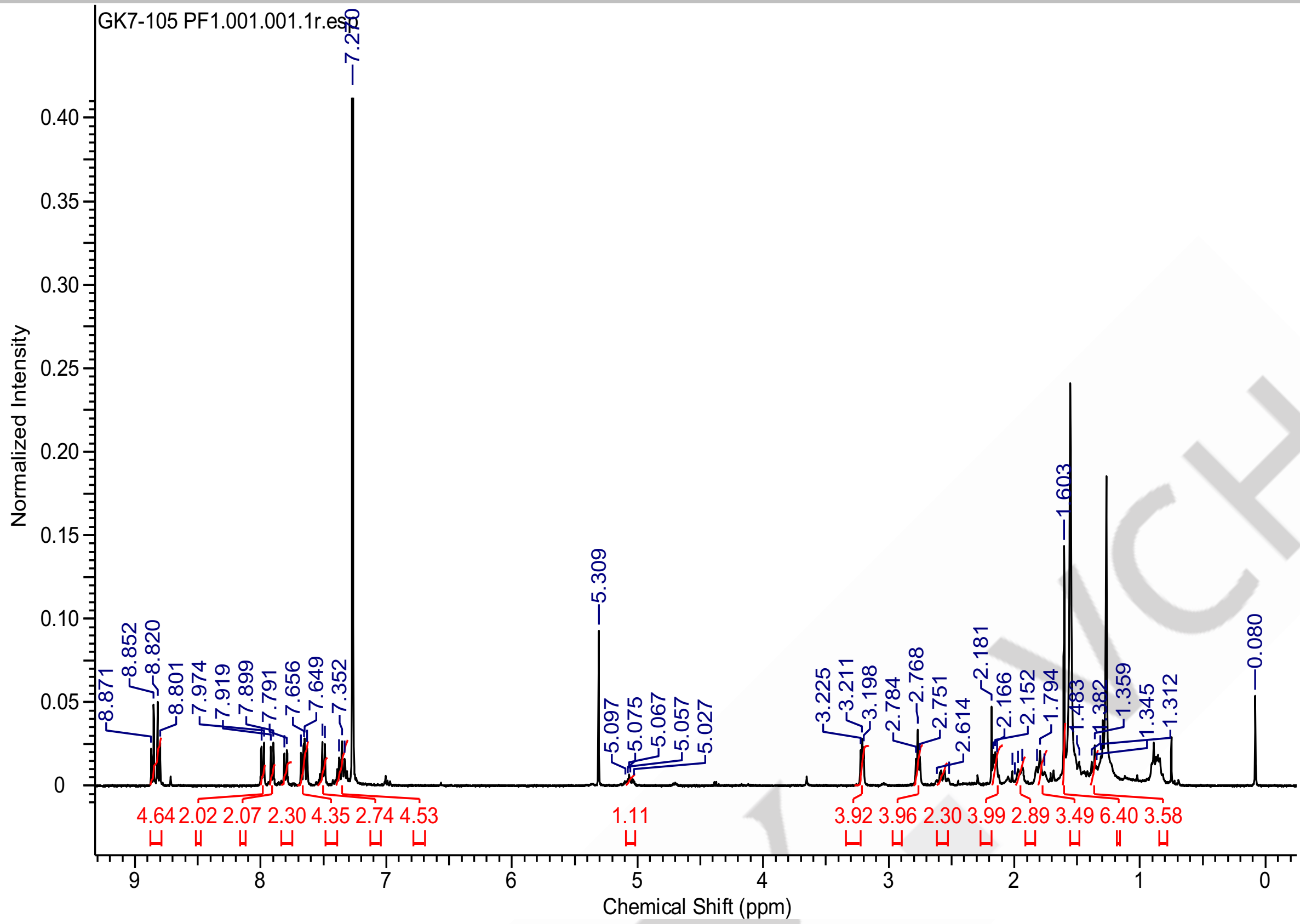

Figure S16: ^{1}H NMR spectrum of **AnthraPol** ($CDCl_3$, 400 MHz)

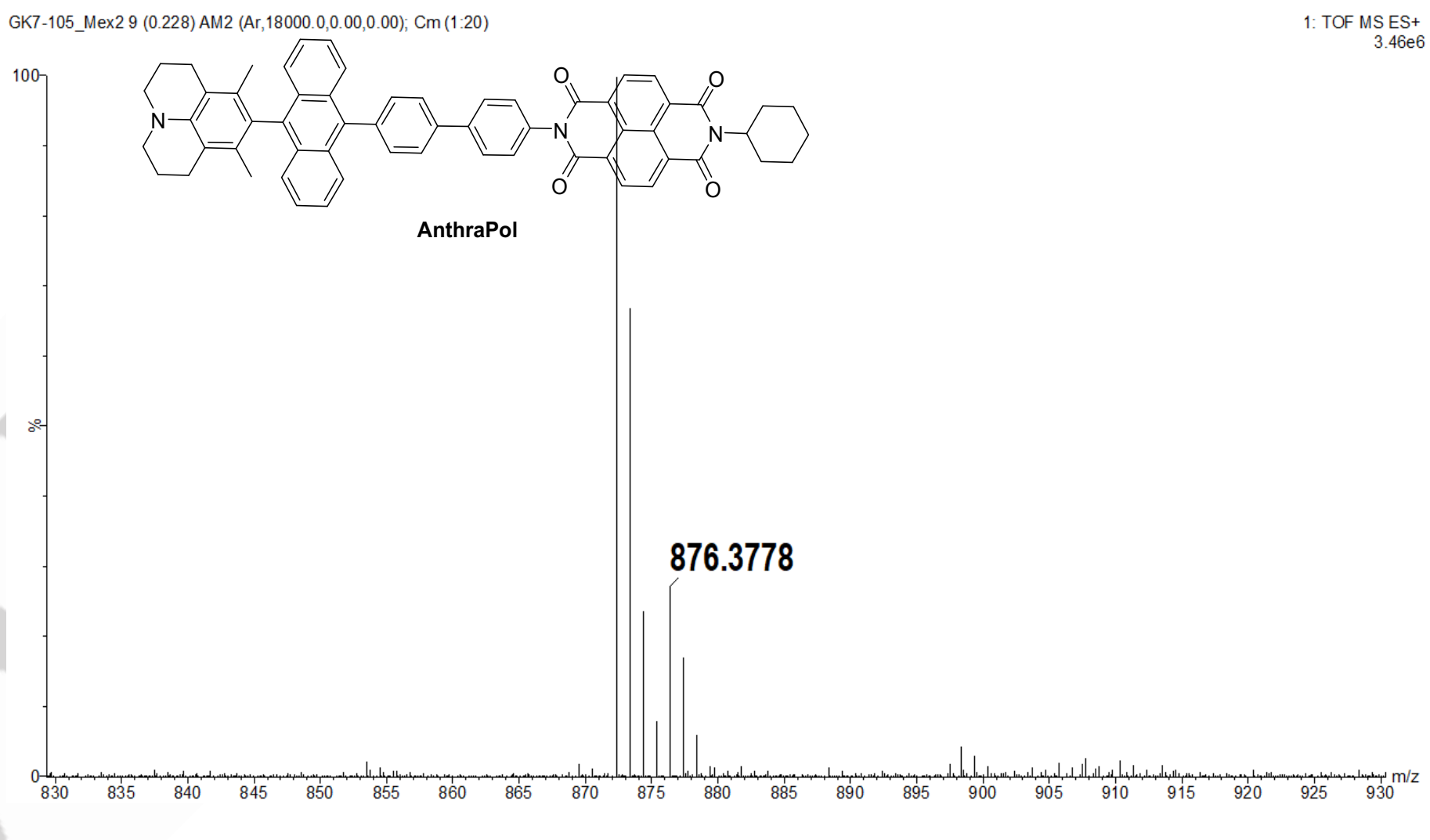

Figure S17: ESI-HRMS spectrum of **AnthraPol**

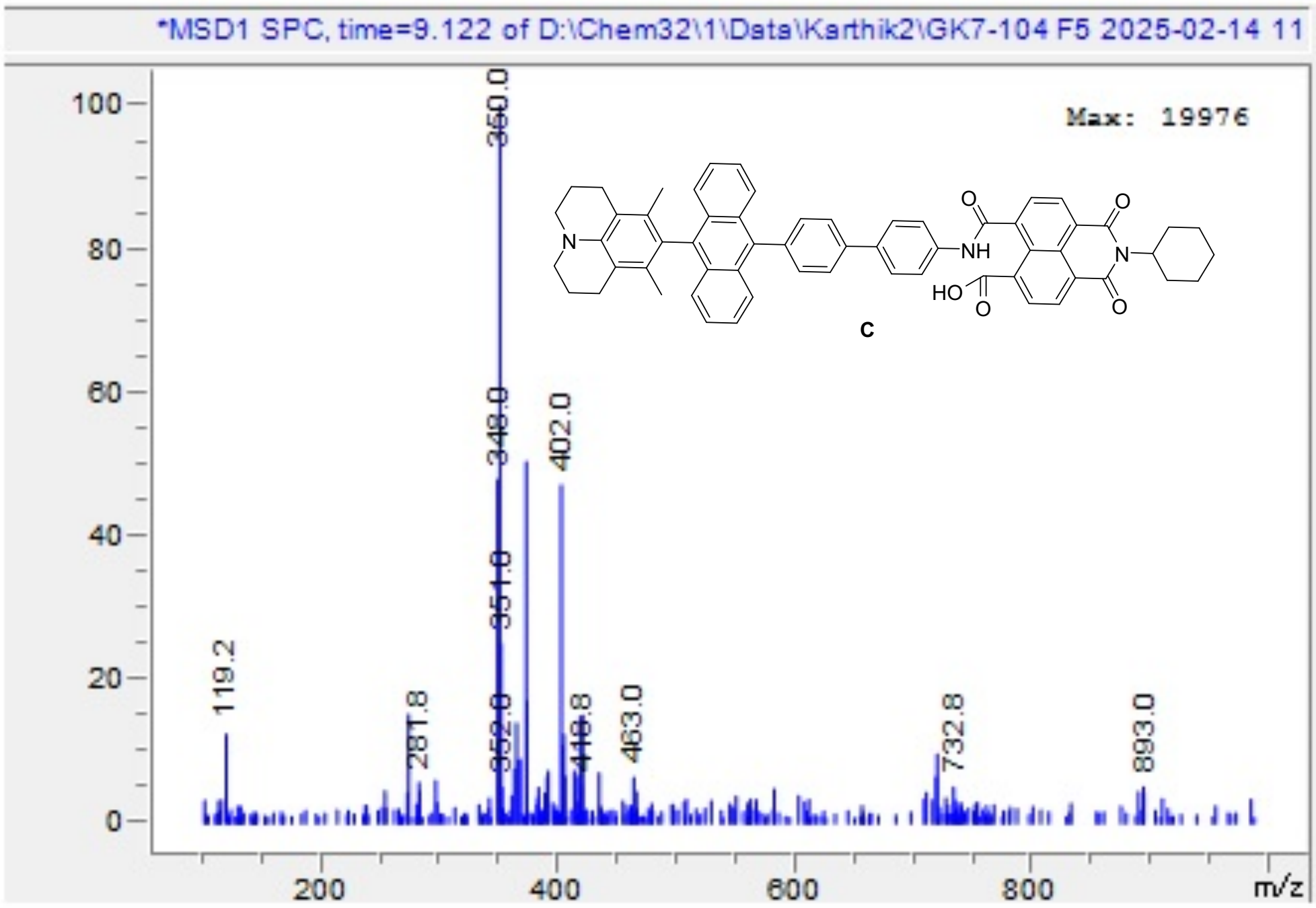

Figure S18: ESI-LCMS spectrum of Intermediate **C**.

S10) References

[1] F. De Biasi, G. Karthikeyan, M. Visegrádi, M. Levien, M. A. Hope, P. J. Brown, M. R. Wasielewski, O. Ouari, L. Emsley, *J. Am. Chem. Soc.* **2024**, *146*, 19667-19672.

[2] F. De Biasi, M. A. Hope, Y. Qiu, P. J. Brown, M. Visegrádi, O. Ouari, M. R. Wasielewski, L. Emsley, *J. Phys. Chem. Lett.* **2024**, *15*, 5488-5494.

[3] F. De Biasi, M. Visegrádi, M. Levien, B. F. Chmelka, L. Emsley, *J. Chem. Phys.* **2025**, *163*, 244113.

[4] P. J. Brown, Y. Qiu, E. I. Latawiec, B. T. Phelan, N. A. Tcyrulnikov, J. R. Palmer, M. D. Krzyaniak, S. M. Kopp, Y. Huang, R. M. Young, M. R. Wasielewski, *J. Phys. Chem. A* **2024**, *128*, 9371-9382.

[5] Z. E. X. Dance, S. M. Mickley, T. M. Wilson, A. B. Ricks, A. M. Scott, M. A. Ratner, M. R. Wasielewski, *J. Phys. Chem. A* **2008**, *112*, 4194-4201.

[6] Z. E. X. Dance, M. J. Ahrens, A. M. Vega, A. B. Ricks, D. W. McCamant, M. A. Ratner, M. R. Wasielewski, *J. Am. Chem. Soc.* **2008**, *130*, 830-832.

[7] J. Gawronski, M. Brzostowska, K. Kacprzak, H. Kolbon, P. Skowronek, *Chirality* **2000**, *12*, 263-268.
